# A Bayesian multivariate factor analysis model for evaluating an intervention by using observational time series data on multiple outcomes

**DOI:** 10.1111/rssa.12569

**Published:** 2021-01-15

**Authors:** Pantelis Samartsidis, Shaun R. Seaman, Silvia Montagna, André Charlett, Matthew Hickman, Daniela De Angelis

**Affiliations:** University of Cambridge, UK; University of Turin, Italy; Public Health England, London, UK; University of Bristol, UK; University of Cambridge, UK

**Keywords:** Causal inference, Factor analysis, Intervention evaluation, Panel data

## Abstract

A problem that is frequently encountered in many areas of scientific research is that of estimating the effect of a non-randomized binary intervention on an outcome of interest by using time series data on units that received the intervention (‘treated’) and units that did not (‘controls’). One popular estimation method in this setting is based on the factor analysis (FA) model. The FA model is fitted to the preintervention outcome data on treated units and all the outcome data on control units, and the counterfactual treatment-free post-intervention outcomes of the former are predicted from the fitted model. Intervention effects are estimated as the observed outcomes minus these predicted counterfactual outcomes. We propose a model that extends the FA model for estimating intervention effects by jointly modelling the multiple outcomes to exploit shared variability, and assuming an auto-regressive structure on factors to account for temporal correlations in the outcome. Using simulation studies, we show that the method proposed can improve the precision of the intervention effect estimates and achieve better control of the type I error rate (compared with the FA model), especially when either the number of preintervention measurements or the number of control units is small. We apply our method to estimate the effect of stricter alcohol licensing policies on alcohol-related harms.

## 1 Introduction

In this work, we consider the problem of estimating the causal effect of an intervention on an outcome of interest in the setting where

(a) the intervention is binary,
(b) assignment of the sample units to the intervention is non-randomized,
(c) only a small number of units are treated and
(d) there are multiple measurements of the outcome both before and after the intervention occurs.

This problem is frequently encountered in various fields of scientific research, including econometrics, epidemiology, marketing, public health and political science. For example, Card (1990) studied the effect that the mass migration in 1980 of Cubans to Miami had on Miami’s labour market, by treating Miami as having received the ‘intervention’ of mass Cuban migration and comparing it with other US states that were not subject to such migrations; Cavallo *et al*. (2013) assessed the effect that large-scale natural disasters, such as earthquakes and storms, had on the gross domestic product of a country by comparing countries that are subject to such natural disasters (the ‘intervention’) with countries not experiencing such disasters; de Vocht (2016) investigated whether the increased use of mobile phones (the ‘intervention’) led to an increase in incidences of certain types of brain cancer in England.

A general difficulty when estimating the causal effect of an intervention from observational data is the potential existence of confounding variables. These are variables which affect both the outcome of interest and the probability of being assigned to the intervention. Failure to account for confounding can lead to biased estimation of causal effects. When the number of units receiving the intervention is large, propensity score methods (Robins *et al*., 2000) can be used. However, when few units receive the intervention, there is not enough information to fit propensity score models. For this reason, several new methodologies for causal inference in the setting where conditions (a)–(d) apply have recently been proposed. For a recent review, see Samartsidis *et al*. (2019).

Many of these methods, including those of Abadie *et al*. (2010), Hsiao *et al*. (2012), Gobillon and Magnac (2016), Chan and Kwok (2016) and Xu (2017), build on the *factor analysis* (FA) model. FA is a natural way to adjust for confounding. The model allows for unobserved confounders that remain constant over time but have a time-varying effect on the outcome. However, current methodologies based on the FA model have shortcomings. Firstly, they can be applied to only a single outcome at a time. When there is more than one correlated outcome, it might be more efficient to model them jointly. Secondly, none of the aforementioned methods explicitly models the temporal correlation between the multiple measurements of the outcome. Modelling auto-correlation may improve efficiency. Thirdly, when the total number of units is small, it is difficult to perform inference for the causal effects by using the existing methods. Finally, some of the approaches above require specifying the number of factors in the model. Although guidance is provided for how to use the data to choose this number, inference using these methods does not account for this data-dependent choice and hence tends to be anticonservative.

In this paper, we attempt to address these shortcomings. We consider extensions of the FA model that can exploit the correlation between different outcomes and the temporal correlation within each outcome, leading to more efficient estimates. Also, by taking a Bayesian approach, we can obtain credible intervals for the causal effects that account for the uncertainty in the number of factors. We contribute to the literature on causal inference in the setting where conditions (a)–(d) apply, in three ways. Firstly, we develop a novel approach that uses multivariate outcomes. An alternative multivariate model was suggested by Robbins *et al*. (2017). However, their method is designed for high dimensional data and its utility in a small data setting is unclear. Secondly, our method is one of the few that model temporal correlation within each outcome. Brodersen *et al*. (2015) recently proposed the *causal impact* method to account for such correlation. However, causal impact can be applied to only a single treated unit and single outcome at a time. Thirdly, our use of the Bayesian approach enables more inference on the causal effects of interest in comparison with other FA-based approaches.

Our method has connections with various applications of FA in contexts other than causal inference. More specifically, this is not the first time that a multivariate factor model has been used in practice. De Vito *et al*. (2018a, b) and Avalos-Pacheco *et al*. (2018) demonstrated the benefits of taking a multivariate approach in genomic applications when dealing with multiple studies rather than multiple outcomes. Assuming a temporal structure in the FA model is also not uncommon; see, for example, McAlinn *et al*. (2019) for a recent application in macroeconomics. However, to our knowledge, this is the first time that either of these extensions (joint outcome modelling and explicit modelling of the temporal correlation) to the standard FA model has been implemented in a causal inference problem and the benefits of using them demonstrated. Our methodology, together with other causal inference methodologies based on FA, is related to the *latent class analysis* (LCA) causal inference approach (Lanza *et al*., 2013; Bartolucci *et al*., 2016; Tullio and Bartolucci, 2019). In LCA, there is a fixed number of classes (the analogue of factors in FA) and the distribution of the outcomes on each unit and at each time point depends on the unobserved class of that unit at that time point. Hence, both LCA and FA attempt to model the variability in the outcome by using latent variables. A difference between LCA and FA is that classes are discrete whereas factors are continuous. Despite being similar in spirit to FA approaches, LCA-based causal inference methods cannot be used in the setting where conditions (a)–(d) apply, mainly because they require estimation of the propensity score, which is problematic when the number of treated units is small. Moreover, these methods focus on the causal effect of the intervention on the probability that an individual belongs to a certain class, whereas in our problem the interest is in the effect of the intervention directly on the outcomes.

The paper is structured as follows. Section 2 introduces our motivating example. Section 3.1 introduces the notation and causal framework. The standard FA model is formulated in Section 3.2. Section 3.3 presents the methodology proposed. Section 3.4 describes how our method accounts for the uncertainty regarding the true number of factors. Prior distributions and posterior sampling are discussed in Section 3.5. Section 3.6 describes how point estimates and inferences are obtained for the causal effects of interest. In Section 4 we perform a series of simulation studies to evaluate the utility of the methodology, using the standard FA model as our benchmark. Section 5 describes the application of methods to our motivating data set. Finally, Section 6 contains a discussion and suggests some possible directions for future research.

The data that are analysed in the paper and the programs that were used to analyse them can be obtained from https://osf.io/4d7c6/.

## 2 Motivating example

Alcohol consumption has an adverse effect on society, being responsible for some harmful health conditions and behaviours. National policy makers have long focused on the development of effective strategies to limit these negative effects. For example, the 2003 Licensing Act (http://www.legislation.gov.uk/ukpga/2003/17/contents) in England and Wales enables local authorities to develop *cumulative impact policies* (CIPs) i.e. to reject automatically new licensing applications unless these are supported by evidence that granting will not negatively impact on surrounding premises.

In a recent study, de Vocht *et al*. (2017) assessed the effect that CIPs had on alcohol-related harms. They collected data on four alcohol-related outcomes: hospital admission rate per 10000 people, violent crimes rate per 1000 people, sexual crime rate per 1000 people and antisocial behaviour incidence rate per 1000 people. The data on each outcome were collected quarterly for the period from mid-2009 to 2015. de Vocht *et al*. (2017) defined intervention sites as local councils implementing a CIP in 2012, and control sites as local councils that did not adopt a CIP at any time during the study period. They identified five treated and 86 control sites in England and Wales.

**Fig. 1:**
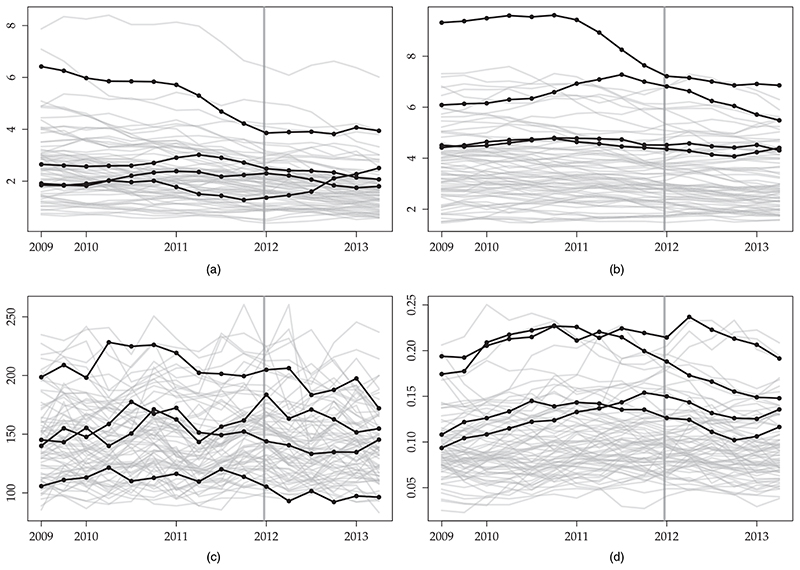
The alcohol licensing data set: each plot shows the time series for each unit for one of the four outcomes (a) antisocial behaviour, (b) violent crimes, (c) hospital admissions and (d) sexual crimes: 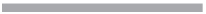, controls; ⦁, treated;|, introduction of the intervention

In Section 5, we demonstrate our proposed methodology by using a subset of data in de Vocht *et al*. (2017). We exclude data from one treated site (Tyneside) because the intervention was implemented earlier in this site and from nine control sites because of missing values in some of the outcomes. Finally, we use data only up to mid-2013, because trends in the following months might be due to changes in the way that crimes were reported (de Vocht *et al*., 2017). Fig. 1 shows the data.

## 3 Model specifications

### 3.1 Notation and causal framework

We have observations y_*itk*_, where *i*=1, …, *n* indexes the units, *t* =1, …, ***T*** indexes the time points and *k* =1, …, ***K*** indexes the outcomes. The units are ordered so that the first *n*_1_ are the controls, i.e. units that do not receive the intervention during the course of the study. For the remaining *n*_2_ =*n*−*n*_1_ units, there is a time point ***T***_1_ after which they all receive the intervention. We refer to these units as the *treated* units. Let *r_i_* be a binary indicator of whether unit i is treated. The study can be split into two periods: the preintervention period consisting of the first ***T***_1_ time points when none of the n units has the intervention, and the post-intervention period consisting of the remaining ***T***_2_ = ***T*** − ***T***_1_ time points when the intervention is in place for the *n*_1_ treated units.

In this paper, we adopt the *Rubin causal model* (Rubin, 1974; Holland, 1986). This means that for each treated unit *i* (*i* > *n*_1_), time *t* after intervention (i.e. *t* > ***T***_1_) and outcome *k* there are two potential outcomes $y_{itk}^{\left(0\right)}$ and $y_{itk}^{\left(1\right)}$; $y_{itk}^{\left(0\right)}$ represents the outcome that would have been observed if the intervention had not been applied and $y_{itk}^{\left(1\right)}$ is the outcome that would be observed if the intervention were applied. Hence, the causal effect of the intervention for any *i* > *n*_1_, *t* > ***T***_1_ and *k* is given by

(1) $$\theta_{itk}=y_{itk}^{\left(1\right)}-y_{itk}^{\left(0\right)}.$$

We are further interested in the average treatment effect on outcome *k* at time *t* in the treated units, *ϑ_tk_*, defined as

(2) $$\vartheta_{tk}=\frac{1}{n_{2}}\overset{n}{\underset{i=n_{1}+1}{\sum }}\theta_{itk}.$$

For treated units before intervention (i.e. *i*>*n*_1_ and *t* ≼ ***T***_1_) and for control units at all times (i.e. *i* ≼ *n*_1_ and all *t*), $y_{itk}^{\left(0\right)}=y_{itk}$ for all *k*, and so is observed. We do not observe $y_{itk}^{\left(0\right)}$ for treated units after intervention, and so causal effects (1) and (2) are not observed. Our approach is to assume a model for $y_{itk}^{\left(0\right)}$, to use this to obtain predictions $y_{itk}^{\left(0\right)}$ for the counterfactuals ${\hat{y}}_{itk}^{\left(0\right)}$ for *i* > *n*_1_ and *t* > ***T***_1_, and then to estimate equations (1) and (2) as ${\hat{\theta }}_{itk}=y_{itk}^{\left(1\right)}-{\hat{y}}_{itk}^{\left(0\right)}$ and ${\hat{\vartheta }}_{tk}=\left(1/n_{2}\right)\sum_{i=n_{1}+1}^{n}{\hat{\theta }}_{itk}$ respectively.

### 3.2 Factor analysis model for a single outcome

For time series observational data, a model that is frequently used for $y_{itk}^{\left(0\right)}$ is the FA model, which is also known as the *interactive fixed effects* model (Bai, 2009). Gobillon and Magnac (2016), Chan and Kwok (2016) and Xu (2017) used the FA model for causal inference in the setting that we are investigating, i.e. that where conditions (a)–(d) apply. Abadie *et al*. (2010) and Hsiao *et al*. (2012) showed that their proposed estimators of the counterfactuals $y_{itk}^{\left(0\right)}$ (*i*>*n*_1_ and *t* > ***T***_1_) are unbiased when the FA model is the data-generating mechanism.

The FA model for the *k*th outcome assumes that

(3) $$y_{itk}^{\left(0\right)}=\gamma_{ik}^{\text{T}}{\text{s}}_{tk}+\varepsilon_{itk},$$

where *γ_ik_* =(*γ_ik1_*, …, *γ_ikp1_*)^T^ is the *p*_1_-vector of unobserved unit-specific loadings for outcome *k*, **s**_*tk*_ =(*s_tk1_*, …, *s_tkp1_*)^T^ ∼*N_p1_*(**0**, **I**) is the *p*_1_-vector of unobserved time-specific factors for outcome *k* and $\varepsilon_{itk}∼N\left(0,\psi_{ik}^{2}\right)$ is the error term. One can view the loadings ***γ***_ik_ as unit characteristics that remain constant over time and the factors **s**_tk_ as their time-varying effect on the potential outcome. Xu (2017) refered to **s**_*tk*_ as ‘shocks’; ***γ***_*ik*_ describe the magnitude of the effect that these shocks have on unit i’s outcome *k*. Variables that are predictive of $y_{itk}^{\left(0\right)}$ but are not affected by the intervention can be incorporated as covariates **x**_it_ in the FA model by replacing equation (3) with

(4) $$y_{itk}^{\left(0\right)}=\gamma_{ik}^{\text{T}}{\text{s}}_{tk}+\beta_{k}^{\text{T}}{\text{x}}_{it}+\varepsilon_{itk}.$$

Such variables may include observed confounders measured before time ***T***_1_. For simplicity, we shall omit such covariates until Section 3.5.

We note in passing that a special case of the FA model is the *difference-in-differences* model (Angrist and Pischke, 2009; Jones and Rice, 2011). This is the FA model with *p*_1_ =2, *s*_*tk*1_ =1 and *γ*_*ik*2_ = 1, i.e. fixed effects for units and time points. The difference-in-differences model assumes that the ‘shocks’ at each time point affect all the units in the same way. This model is frequently used for causal inference with time series observational data.

Recall that the potential outcome $y_{itk}^{\left(0\right)}$ is observed at all times (i.e. *t* =1, …, *T*) for control units (*r_i_* = 0) but at only the preintervention times (i.e. *t* = 1, …, ***T***_1_) for treated units (*r_i_* = 1). Model (3) is fitted to these observed data, considering the post-intervention outcomes $y_{itk}^{\left(0\right)}$ on treated units as missing. The resulting estimator ${\hat{\theta }}_{itk}$ of the intervention effect on unit *i* and outcome *k* at time *t* is asymptotically unbiased as *n*_1_ → ∞ and ***T***_1_ → ∞, provided that *r_i_* is independent of ɛ_*i1k*_, …, ɛ_*iTk*_ (and assuming regularity conditions) (Xu, 2017).

There are two intuitive ways to understand why this asymptotic unbiasedness holds. First, as *n*_1_ and ***T***_1_ become larger (assuming fixed *n* − *n*_1_ and *T* − ***T***_1_), the amount of data for learning about the factors **s**_*tk*_ and the loadings *γ*_ik_ increases, so that the factors and loadings (and hence the expectation of $y_{itk}^{\left(0\right)}$) are increasingly accurately estimated. Second, by letting $y_{tk}^{\left(0\right)}={\left(y_{1tk}^{\left(0\right)},\ldots ,y_{ntk}^{\left(0\right)}\right)}^{T}$, defining **Γ**_*k*_ as the *n*×*p*_1_ matrix with *i*th row *γ_ik_*, and letting ϵ_*tk*_ = (ϵ_1*tk*_, …, ϵ_*Ntk*_)^T^, equation (3) implies that

(5) $${\text{y}}_{tk}^{\left(0\right)}=\Gamma_{k}{\text{s}}_{tk}+\epsilon_{tk},$$

for all *t* and *k*. From this, marginally, i.e. integrating out the factors and error terms, we have that

(6) $$\mathrm{cov}\left(y_{tk}^{\left(0\right)}\right)=\Gamma_{k}\Gamma_{k}^{\text{T}}+\Psi_{k}.$$

where $\Psi_{k}=\text{diag}(\psi_{1k}^{2},\ldots ,\psi_{nk}^{2}).$ Hence, the FA model assumes that the covariance of the potential (treatment-free) outcomes of the n units is the same at all time points. The preintervention data are used to learn about this covariance which is then used to predict the (counterfactual) potential outcomes of the treated units after intervention from the (observed) potential outcomes of the control units after intervention. The larger are *n*_1_ and ***T***_1_, the more information is available to estimate **Γ**_*k*_ and **Ψ**_*k*_, and hence the more accurately we can estimate them (and, from them, the expectation of $y_{itk}^{\left(0\right)}$).

It is worth noting that the FA model allows for a certain form of unmeasured confounding. This is because the aforementioned asymptotic unbiasedness property of ${\hat{\theta }}_{itk}$ does not require *r_i_* to be independent of *γ*_*ik*_. If *γ*_*ik*_ is indeed associated with *r_i_* then, because it is also associated with ${\hat{\theta }}_{itk}$ (see equation (3)), it is an (unobserved) confounder.

### 3.3 Extending the factor analysis model

Our proposed model involves two extensions to the FA model: joint outcome modelling and temporal dependence. We present these two extensions separately, although the model that we finally propose, ‘MVFA+AR’, includes both extensions.

#### 3.3.1 Joint outcome modelling

The classical FA model considers each of the *K* different outcomes independently; it makes no assumptions about correlations between outcomes *k* and *k′* (*k′* ≠ *k*). In situations where the different outcomes are measures of, or are influenced by, a common underlying process, this may be an inefficient way to estimate intervention effects. For example, the outcomes gross domestic product and employment rate can be considered to be two measures of the underlying health of an economy; and rates of hospital admission, violent crime, sexual crime and antisocial behaviour are all influenced by problematic alcohol use. In these situations, part of the variability of the different outcomes is shared. Such shared variability can be modelled by using a multivariate FA model. As we explain below, the multivariate FA model enables the counterfactual post-intervention *k*th outcomes of the treated units to be estimated by using the data on all *K* outcomes, rather than (as in the FA model) just the data on the *k*th outcome. This makes it possible to estimate these counterfactual outcomes—and hence the intervention effects—more precisely.

The multivariate FA model assumes that

(7) $$y_{itk}^{\left(0\right)}=\gamma_{ik}^{\text{T}}{\text{s}}_{tk}+\lambda_{i}^{\text{T}}{\text{f}}_{tk}+\varepsilon_{itk},$$

where *γ*_*ik*_ and **s**_*tk*_ are as defined earlier (i.e. they are unit-specific loadings and time-specific factors, both of which are specific to the *k*th outcome), ***λ***_i_ is the *p*_2_-vector of unit-specific loadings that are shared across outcomes, **f**_*tk*_ ∼ *n*_p2_(**0**, **I**) is a *p*_2_-vector of time-specific factors for ***λ***_i_, and $\varepsilon_{itk}∼N\left(0,\psi_{ik}^{2}\right)$ is the error term. Again, covariates can be included in the model by adding the term $\beta_{k}^{\text{T}}{\text{x}}_{it}$ to the right-hand side of equation (7).

The interpretation of the multivariate FA model follows that of the FA model. More specifically, as well as *γ*_*ik*_, we now have ***λ***_i_, which can be thought of as unit-specific unobserved variables that affect all outcomes; their effect on outcome *k* at time t is quantified by the factor **f**_*tk*_. One way to think about the benefit of jointly modelling the outcomes when estimating the counterfactual outcomes is by appreciating that the joint model learns about ***λ***_i_, the unit-specific loadings that are common to all the outcomes, from the data on all the outcomes. This means that $\gamma_{ik}^{\text{T}}{\text{s}}_{tk}+\lambda_{i}^{\text{T}}{\text{f}}_{tk}$, the expectation of the counterfactual *k*th outcome, is more accurately estimated than in the case when modelling the outcomes independently. An alternative way to think about this benefit is to consider the covariance matrix for $y_{tk}^{\left(0\right)}$. For the FA model, this is given by equation (6). For the multivariate FA model, we have that

$$\mathrm{cov}\left({\text{y}}_{tk}^{\left(0\right)}\right)=\Gamma_{k}\Gamma_{k}^{\text{T}}+{\text{ΛΛ}}^{\text{T}}+\Psi_{k},$$

for each *k* and *t*, where **Λ** is the *n* × *p*_2_ matrix with ith row ***λ***_i_. By modelling the outcomes jointly, this covariance can be estimated more accurately, because the part that is attributable to the shared factors, i.e. **ΛΛ** ^T^, is estimated by using data from all the *K* outcomes. Since this covariance is used to predict the (counterfactual) potential outcomes of the treated units after the intervention, estimating it more accurately should lead to more accurate estimation of those outcomes.

We expect the benefit of jointly modelling the outcomes to be greatest when ***T***_1_ is small. In this situation, there are little data to learn the unit-specific loadings, and so the gain from learning about some of them (specifically ***λ***_i_) by using all the outcomes is likely to be most marked. Also, the greater is the proportion of factors that are common, i.e. the larger the *p*_2_/*p*_1_, the greater is likely to be the benefit from using the multivariate FA model. Note that joint modelling of multiple outcomes should be beneficial in terms of improving the precision of the estimate of the causal effect even when the effect of intervention on only one of the *K* outcomes is of interest. Also note that time-dependent variables that are predictive of ${\text{y}}_{ik}^{\left(0\right)}$ but which are affected by the intervention cannot be included as covariates in the multivariate FA model (as in equation (4)). However, they can be used as additional outcomes in the multivariate FA model.

#### 3.3.2 Modelling temporal dependence

The effect of the unit-specific loading *γ*_*ik*_ (or ***λ***_i_) on the outcomes at times *t* and *t*′ is represented by **s**_*tk*_ and **s**_*t′k*_ (or **f**_*tk*_ and **f**_*t′k*_). It may be reasonable to believe that this effect is likely to be more similar at two nearby times than at two distant times; for example **s**_*tk*_ and **s**_t+1,k_ are likely to be more similar than **s**_*tk*_ and **s**_t+10,k_. Neither the FA nor the multivariate FA model described above takes this time ordering into account. We can take into account the time ordering by assuming that, for each outcome *k*, the factors are generated by an auto-regressive AR(1) process. Specifically, we assume that, for each *k* and *j* = 1, …, *p*_1_ + *p*_2_, we have that

(8) $$s_{tkj}=\rho_{kj}s_{t-1,kj}+\eta_{tkj},$$

where *s_tkj_* = *f*_*tk*,*j*−*p*1_ for *p*_1_ < *j* ≤ *p*_1_ + *p*_2_, *ρ_kj_* ∈ (−1, 1) are persistent parameters and *η_tkj_* ∼ *N*(0, 1).

Assuming that factors are generated by an AR(1) process may improve prediction of the counterfactual outcomes $y_{itk}^{\left(0\right)}$ (*i*>*n*_1_, *t*>***T***_1_) and hence increase the precision of the intervention effect estimates. This can become clear as follows. By integrating out the factors and error terms, we find that, for *t′* ≠ *t*

(9) $$\mathrm{cov}\left({\text{y}}_{tk}^{\left(0\right)},{\text{y}}_{t^{\prime }k}^{\left(0\right)}\right)={\text{Γ}}_{k}\mathrm{cov}\left({\text{s}}_{tk},{\text{s}}_{t^{\prime }k}\right){\text{Γ}}_{k}^{\text{T}}+\text{Λ}\mathrm{cov}\left({\text{f}}_{tk},{\text{f}}_{t^{\prime }k}\right){\text{Λ}}^{\text{T}},$$

Equation (9) shows that, by assuming an AR(1) prior for the factors, an *a priori* correlation both between *y_itk_* and *y_it′k_* is allowed, as well as between *y_itk_* and *y_i′ t′k_*, where *i* ≠ *i′*. If these correlations are strong, the sharing of information across time points can lead to more accurate estimates of the counterfactuals. This does not happen in the standard FA model which assumes that *ρ_kj_* = 0, and therefore the right-hand side of equation (9) reduces to **0**.

We expect that assuming an AR structure for the factors will increase efficiency in settings where *n*_1_ is small and ***T***_1_ large. In these settings, there are few observations per time point and therefore factors cannot be estimated accurately. By assuming an AR(1) structure, we allow for the sharing of information between nearby time points. When ***T***_1_ is small, there may be less advantage, because there is then less information to estimate *ρ*_kj_.

We call the FA model with this AR(1) structure ‘FA+AR’ and the multivariate FA model with AR(1) structure MVFA+AR.

### 3.4 Choosing the number of factors

One of the challenges when implementing FA is choosing the total number of factors in the model. Many researchers have proposed solutions for this problem; for example, Bai and Ng (2002) proposed some criteria to choose the number of factors; Lopes and West (2004) developed a reversible jump Markov chain Monte Carlo (MCMC) algorithm to estimate the number of factors; Carvalho *et al*. (2008) took an evolutionary stochastic model search approach; Srivastava *et al*. (2017) used a continuous shrinkage prior on the loadings. In this work, we account for the uncertainty in *p*_1_ and *p*_2_ by assuming a *multiplicative gamma process shrinkage* (MGPS) prior (Bhattacharya and Dunson, 2011) on the loadings.

We use the MGPS prior for both the outcome-specific loadings *γ*_*ik*_ and shared loadings ***λ***_i_. We shall describe how this prior works for the outcome-specific loadings; for the shared loadings, the specifications are analogous. Let the loading vector *γ*_*ik*_ be of dimension *p*_1_ = ∞. MGPS assumes that for each j = 1, …, ∞ we have

(10) $$\gamma_{ikj}∼N\left(0,\frac{1}{\phi_{ikj}\tau_{kj}}\right),$$

where *ϕ_ikj_* and *τ_kj_* (both greater than 0) are the local and global shrinkage parameters respectively, such that

(11) $$\tau_{kj}=\overset{j}{\underset{l=1}{\prod }}\delta_{kj}.$$

For appropriately chosen priors on *ϕ*_*ikj*_ and *δ*_*kj*_, the product *ϕ*_*ikj*_ *τ*_*kj*_ increases, thus encouraging the magnitude of the elements of *γ*_*ik*_ to decrease progressively towards 0. Hence, although the number of columns in each matrix **Γ**_*k*_ is infinite there will be a column such that all columns after this column have an *L*_1_-norm of almost 0, indicating that no more factors are required for the data set under consideration.

In practice, it is not possible to carry out computations when loadings are infinite dimensional. So, we let *γ*_*ik*_ be of dimension k_1_ (and *k*_2_ for the shared loadings), where *k*_1_ is sufficiently large.

This approach can be computationally wasteful when *p*_1_ is much smaller than the specified *k*_1_. However, one can easily detect this through a pilot run of the algorithm that is used to simulate from the posterior; if most of the columns of **Γ**_ik_ have an *L*_1_-norm that is very low, then it is recommended to decrease *k*_1_ in the final run. Alternatively, an adaptive way to determine *k*_1_ was discussed by Bhattacharya and Dunson (2011).

The MGPS prior can be used to perform inference on the number of factors. At iteration *l* of the MCMC algorithm (which we use to draw samples from the posterior), let *d* ^(*l*)^ denote the total number of columns in **Γ**_*k*_ whose absolute elements |*γ*_*1kj*_|, …, |*γ*_*nkj*_| are all below a prespecified threshold m. The effective number of factors at iteration *l* is *k*_1_ − *d* ^(*l*)^. Therefore, one can use the posterior distribution of *k_1_ − d* ^(*l*)^ to estimate the total number of factors in the model (e.g. as the posterior median of this distribution) and to construct credible intervals (by using the quantiles). This approach is sensitive to the choice of threshold *m*.

The reasons that we use the MGPS prior instead of the other methods that we mention are twofold. Firstly, MGPS allows for a conjugate formulation of the model, which simplifies posterior sampling. Secondly, it has been shown that this method performs well in a wide range of applications (e.g. Montagna *et al*. (2012), Montagna, Irincheeva and Tokdar (2018) and Montagna, Wager, Barrett, Johnson and Nichols (2018)).

### 3.5 Prior distributions and Markov chain Monte Carlo algorithm

The prior distributions are as follows. For all *i* and *k*, we let the variance parameters $\psi_{ik}^{2}∼$ InverseGamma(0.001, 0.001). For the AR parameters, *ρ*_*kj*_ ∼uniform(−1, 1) for all *k* and *j*. For the shrinkage parameters, we follow recommendations by Bhattacharya and Dunson (2011) and let *ϕ*_*ikj*_ ∼ gamma$\left(\frac{3}{2},\frac{3}{2}\right)$ for all *i*, *k* and *j*, *δ*_*k1*_ ∼ gamma(2:1, 1) for all *k*, and *δ*_*kj*_ ∼ gamma(3:1, 1) for *j* > 1. If covariates **x**_*it*_ are included in MVFA+AR, we let the regression coefficients ***β***_*k*_ ∼ *N*(0, 10^3^ **I**) for all *k*.

The posterior distribution resulting from the MVFA+AR model of equations (7), (8), (10) and (11) and the prior distributions that are stated in this section is analytically intractable. We therefore use MCMC sampling to draw samples from it. In particular, we propose a hybrid Gibbs sampler where each parameter (or block of parameters) is sampled from its full conditional given the remaining parameters, using either Gibbs or Metropolis–Hastings steps. The main challenge is to simulate from high dimensional normal full conditionals. More specifically, the vector of factors ${\text{f}}_{k}={\left({\text{s}}_{1k}^{\text{T}},{\text{f}}_{1k}^{\text{T}},\ldots ,{\text{s}}_{Tk}^{\text{T}},{\text{f}}_{Tk}^{\text{T}}\right)}^{\text{T}}$ for each outcome is drawn from a *T(k*_1_ +*k*_2_)-dimensional normal distribution, and the vector of loadings ${\tilde{\lambda }}_{i}={\left(\lambda_{i}^{\text{T}},\gamma_{i1}^{\text{T}},\ldots ,\gamma_{iK}^{\text{T}}\right)}^{\text{T}}$ for each unit is drawn from a (*Kk_1_ + k*_2_)-dimensional normal distribution. We perform both these updates with good computational efficiency by using the method of Rue (2001). The update of AR hyperparameters *ρ*_*jk*_ is also challenging, because these parameters have bounded support and it is not possible to simulate them directly from their full conditionals. To overcome this issue, we update *ρ*_*jk*_ with Metropolis– Hastings steps by using the proposals that were developed by Kastner and Frühwirth-Schnatter (2014). The remaining model parameters ***β***_*k*_, *ϕ*_*ikj*_, *δ*_kj_ and $\psi_{ik}^{2}$ can be easily drawn from their full conditionals. For full details of the MCMC algorithm, see section A of the web-based supplementary material, where we also provide a sketch of the sampler. Similar MCMC algorithms can be used to draw from the posterior distribution of the FA, FA+AR and MVFA models.

We emphasize that the factors and loadings are not identifiable. Since we are not interested in interpreting these parameters but only in the counterfactuals (which are identifiable), we choose not to impose any identifiability constraints. Users who are interested in interpreting these parameters can resort to one of the existing approaches for ensuring identifiability; see for example section 12.1.3 of Murphy (2012) for a fairly recent overview. One method is to restrict the loading matrix to the class of lower diagonal matrices (Geweke and Zhou, 1996).

### 3.6 Point estimation and inference

Samples from the posterior distribution are used to obtain samples from the posterior distribution of the causal effect *θ*_*itk*_. First, we simulate from the posterior predictive distribution of the counterfactual outcomes

(12) $$y_{itk}^{\left(0,l\right)}={\left(\gamma_{ik}^{\left(l\right)}\right)}^{\text{T}}{\text{s}}_{tk}^{\left(l\right)}+{\left(\lambda_{i}^{\left(l\right)}\right)}^{\text{T}}{\text{f}}_{tk}^{\left(l\right)}+\varepsilon_{itk}^{\left(l\right)}\;\left(i>n_{1},t>T_{1}\right)$$

where $\varepsilon_{itk}^{\left(l\right)}∼N\left\{0,{\left(\psi_{ik}^{2}\right)}^{\left(l\right)}\right\}$ and *l* indexes the MCMC draw. Then, samples $\theta_{itk}^{\left(l\right)}$ from the posterior of the individual effect *θ*_*itk*_ are readily available as $\theta_{itk}^{\left(l\right)}=y_{itk}-y_{itk}^{\left(0,l\right)}$. Similarly, samples $\vartheta_{tk}^{\left(l\right)}$ from the posterior of the average treatment effect *ϑ*_*tk*_ are obtained as $\vartheta_{tk}^{\left(l\right)}=\left(1/n_{2}\right)\sum_{i=n_{1}+1}^{n}\theta_{itk}^{\left(l\right)}$. We can use these to calculate point estimates and to perform inference. For instance, the point estimate of *ϑ*_*tk*_ will be (1/*L*)${\text{Σ}}_{l=1}^{L}\vartheta_{tk}^{\left(l\right)}$, where *L* is the number of MCMC samples and the 95% credible interval for *ϑ*_*tk*_ will be given by the 2.5% and 97.5% percentiles of the $\vartheta_{tk}^{\left(l\right)}$. To test for a positive intervention effect, one can estimate the posterior probability that *ϑ*_*tk*_ >0 as (1/*L*)${\text{Σ}}_{l=1}^{L}⫾\left(\vartheta_{tk}^{\left(l\right)}>0\right)$, where ⫾(·) is the indicator function.

## 4 Simulation studies

### 4.1 Setting

We performed a series of simulation studies to answer the question of whether we can obtain estimates of *ϑ*_*tk*_ that are more precise than those obtained from the standard FA model by

(a) modelling multiple outcomes jointly,
(b) assuming an AR(1) structure for the factors and
(c) doing both simultaneously.

Each data set (from a total of 10000) was simulated as follows. We used MVFA+AR to generate data on *n* = 35 units with ***T***_1_ = 40 preintervention and ***T***_2_ = 5 post-intervention time points. There were *K* =3 outcomes, *p*_1_ =2 outcome-specific loadings and *p*_2_ =4 shared loadings, and the persistent parameters of the factors were *ρ*_*kj*_ = 0:9 for all *k* and *j*. (We set the variance of the error terms *η*_*tkj*_ in equation (8) to 1/(1−*ρ*_*jk*_/for all *k* and *j*, so that *s_tkj_* ∼*N*(0, 1) for all *t*, *j* and *k*.) *s*_0*kj*_ were drawn from an *N*(0, 1) distribution. For each *k*, *i* and *j*, we drew the loadings from an *N*(0, 1) distribution. Finally, for each *k* and *i*, we set $\psi_{ik}^{2}=\frac{1}{3}$.

We randomly chose *n*_2_ = 5 treated units from these 35 units by using the expected values on the first outcome. To introduce unobserved confounding, each unit had selection probability proportional to

$$\text{expit}\left\{\kappa \overset{45}{\underset{t=41}{\sum }}\left(\gamma_{i1}^{\text{T}}{\text{s}}_{t1}+\lambda_{i}^{\text{T}}{\text{f}}_{t1}\right)\right\}$$

of being selected to be a treated unit, where expit(·)=exp(·)/{1+exp(·)}. The value of *κ* controls the degree of unobserved confounding; *κ* = 0 means no unobserved confounding, and *κ* > 0 means that units with larger expected values of the post-intervention (possibly counterfactual) treatment-free first outcome are more likely to be treated. We chose *κ* = 0.75 because we found that a simple t-test comparing $Y_{1}=\left\{y_{1,41,1}^{\left(0\right)},\ldots ,y_{30,41,1}^{\left(0\right)}\right\}$ and $Y_{2}=\left\{y_{31,41,1}^{\left(0\right)},\ldots ,y_{35,41,1}^{\left(0\right)}\right\}$ had a roughly 17.5% rejection rate (this would be around 5% if the elements of *Y*_1_ and *Y*_2_ were exchangeable). This procedure gave us data sets of *n*_1_ =30 control units and *n*_2_ =5 treated units (set-up I), and ***T***_1_ = 40 and ***T***_2_ = 5.

We expected the answers to questions (a)–(c) to depend on ***T***_1_ and *n*_1_. To obtain data sets with fewer than 40 preintervention time points and/or fewer than 30 control units, we discarded the data in the first 40 − ***T***_1_ preintervention time points and/or randomly discarded 30 − *n*_1_ control units. The values of (***T***_1_, *n*_1_) in set-ups II–IX are (40, 30), (40, 15,),, (40, 5), (20, 30), (20, 15), (20, 5), (10, 30), (10, 15) and (10, 5) respectively. The total number of treated units (*n*_2_ = 5) and post-intervention observations (***T***_2_ = 5) were common to all set-ups.

The point estimate of the *ϑ*_*tk*_ is

$${\hat{\vartheta }}_{tk}=\frac{1}{n_{2}}\overset{n}{\underset{i=n_{1}+1}{\sum }}\left(y_{itk}^{\left(1\right)}-{\hat{y}}_{itk}^{\left(0\right)}\right)=\vartheta_{tk}+\frac{1}{n_{2}}\overset{n}{\underset{i=n_{1}+1}{\sum }}\left(y_{itk}^{\left(0\right)}-{\hat{y}}_{itk}^{\left(0\right)}\right).$$

So, if the average (over simulated data sets and over treated units) value of ${\hat{y}}_{itk}^{\left(0\right)}$ (*i* > *n*_1_ and *t* > ***T***_1_) is equal to the average (over simulated data sets and over treated units) of $y_{itk}^{\left(0\right)}$, then ${\hat{\vartheta }}_{tk}$ will be unbiased for any value of *θ*_itk_. Similarly, if the credible interval for $\sum_{i=n_{1}+1}^{n}y_{itk}^{\left(0\right)}$ contains the true value of this sum, then the credible interval for *ϑ*_*tk*_ will also contain the true value of the *ϑ*_*tk*_ for any *θ*_*itk*_. Thus, it sufficed to study the case where *θ*_*itk*_ = 0.

We fit the following models to all data sets: FA, FA+AR, MVFA and MVFA+AR. Note that all these models were correctly specified for the data that we generated. For all methods, we ran the MCMC algorithm for 31250 iterations, applied a thinning factor of 25 to obtain a total of 1250 posterior draws, discarded the first 250 as a burn-in and used the remaining 1000 for inference. FA and FA+AR are designed for univariate outcomes and hence we applied these to each of the outcomes in turn. For all models, we used the MGPS prior, setting *k*_1_ = *k*_2_ = 12.

We used the posterior mean as the point estimate of the *ϑ*_*tk*_; credible intervals were obtained by using the 2.5% and 97.5% quantiles of the posterior distribution.

We compared the performance of the models in terms of the bias and standard error of the point estimates for the *ϑ*_*tk*_s, and mean width and false positive rate of the 95% credible intervals of the *ϑ*_*tk*_s. As a measure of ‘power’, we used the probability of detecting an intervention effect when the true *ϑ*_*tk*_ was not equal to 0. Here, we defined ‘detection’ as a credible interval that excludes zero. For convenience, we assumed that *θ*_*itk*_ was the same for all units, times and outcomes.

### 4.2 Results

**Fig. 2:**
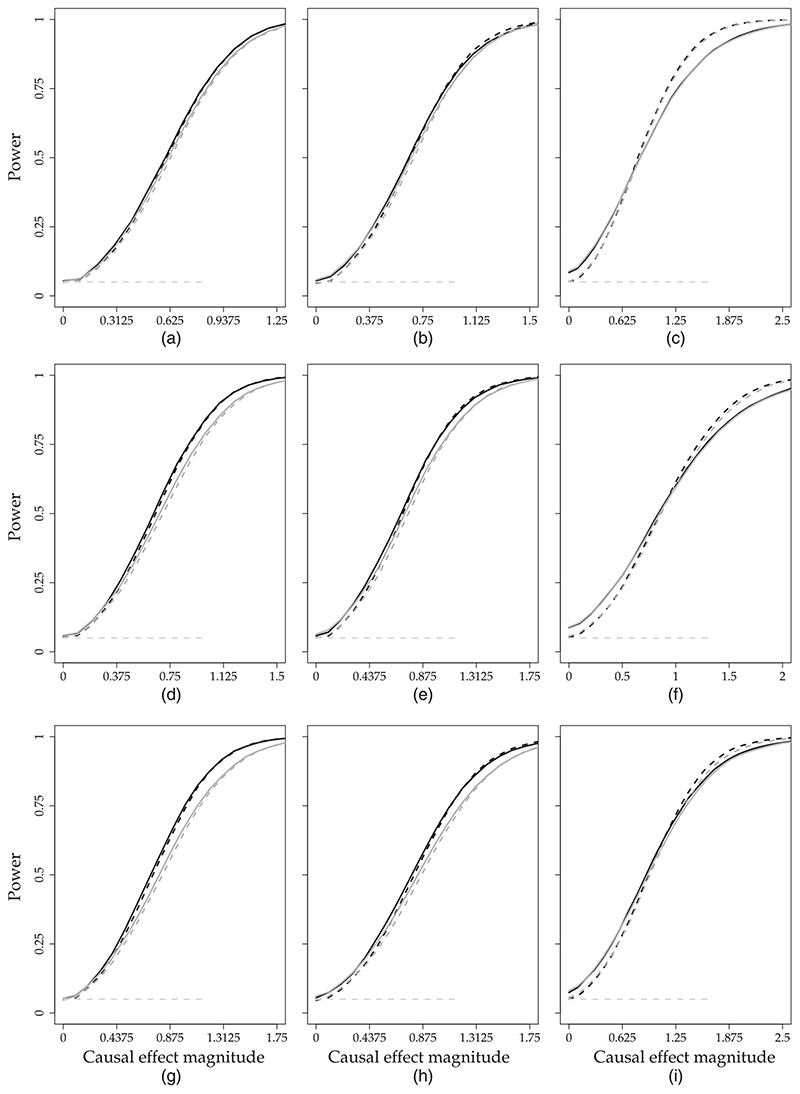
Results of the simulation study for the *first outcome* *k* = 1 and *first post-intervention time point t* = *T*_1_ + 1 (the figure presents the probability of detecting an intervention effect (*y*-axis) as a function of *ϑ_T_*_1_+1,1 (*x*-axis) in set-ups I–IX; all results are based on 10000 data sets simulated from MVFA+AR 
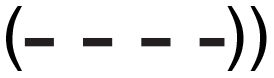
 
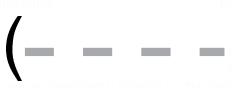
, 5%, the desired detection rate when the intervention *ϑ_T_*_1_+1,1 = 0; 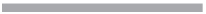, MVFA; 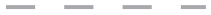, FA+AR; 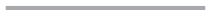, FA): (a) set-up I, *T*_1_ = 40, *n*_1_ = 30; (b) set-up II, *T*_1_ = 40, *n*_1_ = 15; (c) set-up III, *T*_1_ = 40, *n*_1_ = 5; (d) set-up IV, *T*_1_ = 20, *n*_1_ = 30; (e) set-up V, *T*_1_ = 20, *n*_1_ = 15; (f) set-up VI, *T*_1_ = 20, *n*_1_ = 5; (g) set-up VII, *T*_1_ = 10, *n*_1_ = 30; (h) set-up VIII, *T*_1_ = 10, *n*_1_ = 15; (i) set-up IX, *T*_1_ = 10, *n*_1_ = 5

**Fig. 3:**
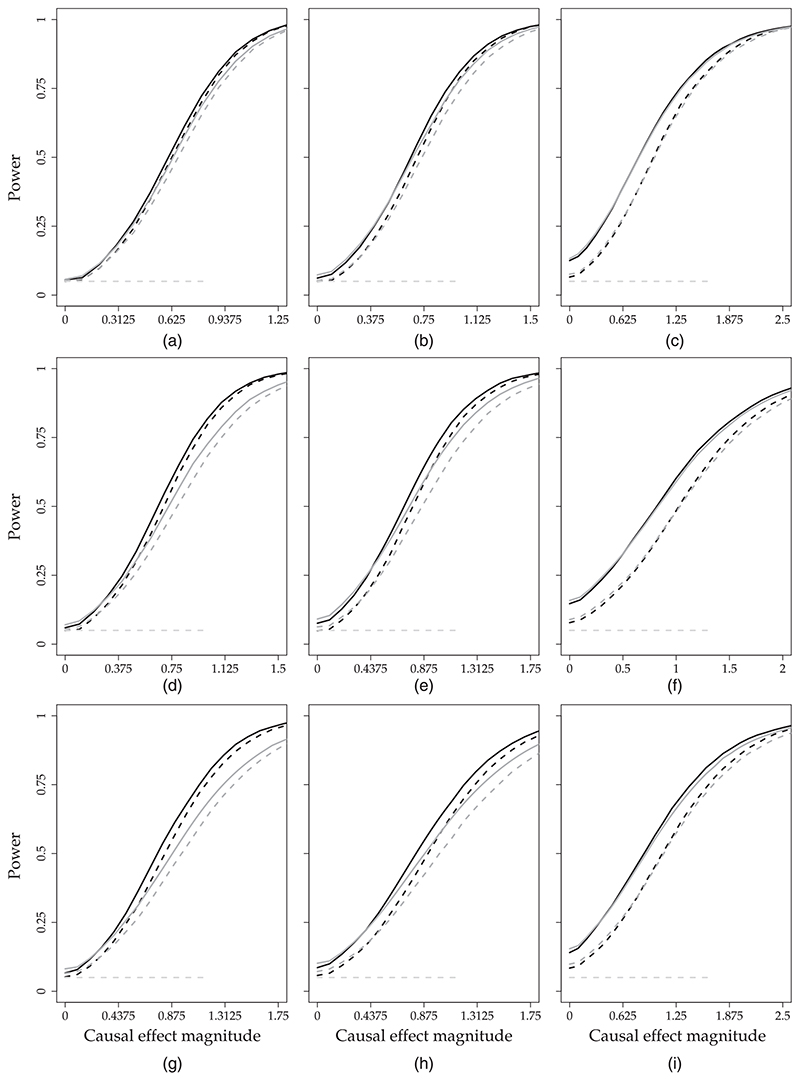
Results of the simulation study for the *first outcome k* = 1 and *last post-intervention time point t* = *T* (the figure presents the probability of detecting an intervention effect (*y*-axis) as a function of *ε_T_*_,1_ (*x*-axis) in set-ups I–IX; all results are based on 10000 data sets simulated from MVFA+AR 
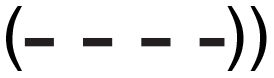
 
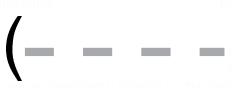
, 5%, the desired detection rate when the intervention *ε_T_*_,1_ = 0; 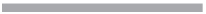, MVFA; 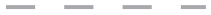, FA+AR; 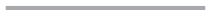, FA): (a) set-up I, *T*_1_ = 40, *n*_1_ = 30; (b) set-up II, *T*_1_ = 40, *n*_1_ = 15; (c) set-up III, *T*_1_ = 40, *n*_1_ = 5; (d) set-up IV, *T*_1_ = 20, *n*_1_ = 30; (e) set-up V, *T*_1_ = 20, *n*_1_ = 15; (f) set-up VI, *T*_1_ = 20, *n*_1_ = 5; (g) set-up VII, *T*_1_ = 10, *n*_1_ = 30; (h) set-up VIII, *T*_1_ = 10, *n*_1_ = 15; (i) set-up IX, *T*_1_ = 10, *n*_1_ = 5

The results for the first outcome (*k* = 1) are summarized in Table 1 and Figs 2 and 3. Table 1 presents the bias, standard error, mean credible interval width and false positive rate for (*k*, *t*)= (1, ***T***_1_ + 1) and (*k*, *t*) = (1, *T*). Figs 2 and 3 show power for (*k*, *t*) = (1, ***T***_1_ + 1) and (*k*, *t*) = (1, *T*) respectively. In section B of the web-based supplementary material, we present results for (*k*, *t*)= (2, ***T***_1_ + 1) (Table 1 and Fig. 1), and for (*k*, *t*) = (3, *T*) (Table 1 and Fig. 2).

To answer question (a), we compare the results that were obtained from MVFA+AR and MVFA with the results that were obtained from FA+AR and the FA model respectively. In settings where ***T***_1_ <40 and *n*_1_ ≽ 15 (i.e. set-ups IV, V, VII and VIII), we see that joint modelling of outcomes leads to considerable gains in precision: MVFA+AR and MVFA decrease the standard error of the point estimates and the mean credible interval width in these settings (see Table 1 and Table 1 of the web-based supplementary material section B). The gains in efficiency are also apparent from the power: Figs 2 and 3 and 1 and 2 of web-based supplementary material section B show that the use of a multivariate model instead of a univariate model can substantially improve the chance of detecting an intervention effect. For example, for (*k*, *t*) = (1, *T*) and set-up VII, we find that, when *ϑ*_T1+1,1_ = 1:2, the intervention effect is detected by MVFA with probability 78% whereas it is detected by the FA model with probability 66%. The power curves for (*k*, *t*)=(2, ***T***_1_ +1) and (*k*, *t*)=(3, T) (web-based supplementary material section B) reveal a similar pattern. We find no settings in which the univariate models outperform the corresponding multivariate models for any of the performance measures that we consider.

To answer question (b), we compare the results that were obtained from MVFA+AR and FA+AR with those obtained from MVFA and the FA model respectively. We find that the inclusion of the AR component leads to either improved or unchanged performance. The improvements occur mainly in settings where *n*_1_ = 5 (i.e. set-ups III, VI and IX). In these settings with few control units, the FA and MVFA models perform very poorly in terms of bias and false positive rate for outcome k = 1 (see Table 1). FA+AR and MVFA+AR offer significant improvements in terms of both bias and false positive rate compared with the FA and MVFA models. For example, for (*k*, *t*) = (1, *T*) and set-up VI, the false positive rate is 8.9% for the FA model whereas it is 5.7% for MVFA+AR. Note that, for outcomes *k* = 2 and *k* = 3, the bias and false positive rate in set-ups III and VI are not as high as for outcome *k* = 1 (see Table 1 of web-based supplementary material section B). The reason is that we have chosen the treated units by using the expected outcomes on *k* =1 and therefore the effect of confounding is greater for *k* =1. The inclusion of the AR component also leads to gains in efficiency in set-ups III and VI, as it reduces both the standard error of the point estimates and the mean credible interval width (Table 1). The gains in power can be moderate. For example, for (*k*, *t*) = (1, *T*) and set-up III, the intervention effect is detected with probability 90% by FA+AR and 83% by the FA model when *ϑ*_T_1_+1,1_ = 1.5 (Fig. 2). The improvement in power is more prominent for outcome *k* = 2 (because the bias of all methods is close to 0 for this outcome). For instance, in set-up III, a *ϑ*_T_1_+1,3_ = 1.5 is detected with probability 86% by FA+AR and 72% by the FA model (Fig. 1 of web-based supplementary material section B).

We find no set-ups in which MVFA+AR performs better than both MVFA and FA+AR. The reason is that, as we explained earlier in Section 3.3, the two proposed extensions (joint outcome modelling and the AR(1) prior on factors) improve on FA in very different settings: the former when ***T***_1_ is small and *n*_1_ is large; the latter when ***T***_1_ is large and *n*_1_ is small. In contrast, we find no settings where either FA+AR or MVFA outperforms MVFA+AR. Therefore, the advantage of MVFA+AR is that it can be used in all settings. For this reason, we suggest that this is the model that should be used in practice.

The gains in efficiency that are obtained by using either FA+AR, MVFA and MVFA+AR will depend not only on ***T***_1_ and *n*_1_ but also on the total number of outcomes K, the number of factors *p*_1_ + *p*_2_ and the ratio *p*_1_/*p*_2_. As *k* increases, ***λ***_i_ will be estimated with higher precision. As the total number of factors *p*_1_ +*p*_2_ increases, the total number of model parameters that need to be estimated increases. Hence, sharing of information (either between outcomes by using MVFA or MVFA+AR or between time points by using FA+AR or MVFA+AR) is more important for larger values of *p*_1_ + *p*_2_. Finally, MVFA and MVFA+AR are well suited to applications where the ratio *p*_1_/*p*_2_ is low, i.e. where the number of shared loadings is large compared with the number of outcome-specific loadings.

## 5 Application to the motivating data set

### 5.1 Model details

In this section we apply the proposed methodology to the alcohol licensing data set that was introduced in Section 2. The data set consists of data on *k* = 4 outcomes relating to the harms of alcohol consumption in society. For each outcome, there are *n*_1_ = 72 control and *n*_2_ = 4 treated units. There are T = 16 observations per unit per outcome, ***T***_1_ = 10 of which are in the preintervention period. The objectives of the analysis are threefold. Firstly, we are interested in assessing the evidence for the existence of common factors underlying the four outcomes. Secondly, we are aiming to investigate the effect of the intervention on each of the four treated units (Derby, Enfield, Kingston upon Thames and Southwark) individually. Thirdly, we wish to assess the evidence for a non-zero average intervention effect *ϑ*_*tk*_ (*t* > 10).

To achieve these goals, we fit our proposed model MVFA+AR. We set *k*_1_ = 20 and *k*_2_ = 10. We run the MCMC algorithm for 1500000 iterations (this took approximately 9 h on a Linux machine with an Intel i7-6700 3.4-GHz central processor unit); the first 250000 samples are discarded as a burn-in and we apply a thinning factor of 500 to the remaining draws. Therefore our MCMC sample consists of 2500 draws from the joint posterior of the model parameters. Convergence is assessed by visual inspection of posterior trace plots for some randomly chosen shrinkage parameters *δ_jk_*, variance parameters $\psi_{ik}^{2}$ and the counterfactuals $y_{itk}^{\left(0\right)}$ (*i* > 72 and *t* > 10). These indicate that the chain has reached its stationary distribution. Further, we run an additional nine chains and compare the posterior densities of these parameters with those obtained from the first chain. No major differences are found. Therefore, we conclude that the chain has converged.

For each outcome, we also fit the univariate FA model with the MGPS prior and k_1_ = 20. However, the conclusions that we reached regarding the effect of the intervention are very similar (except for minor losses in precision of the causal effect estimates) to those obtained from MVFA+AR. Thus, the results from the FA model are not discussed further.

### 5.2 Results

**Fig. 4:**
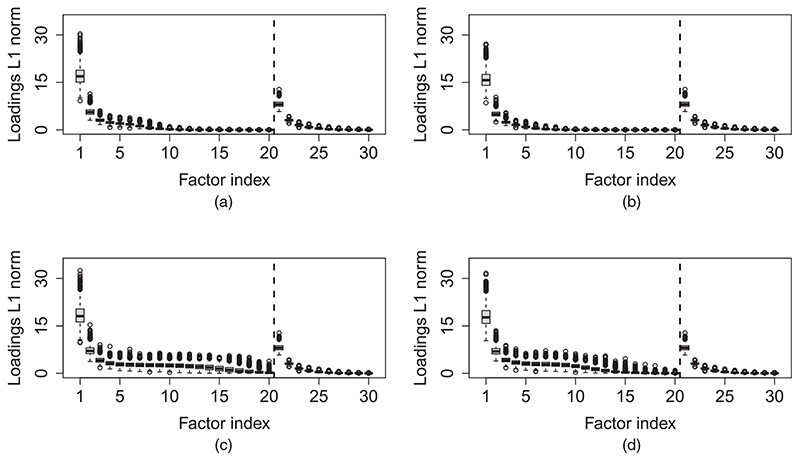
Analysis of the alcohol licensing data set by using the proposed MVFA model (the figure presents posterior boxplots of $\sum_{i=1}^{n}\left|\gamma_{ikj}\right|$, the *L*_1_-norm of the *j*th column of the loadings matrix; the boxplots are based on an MCMC sample of size 2500; factors 1–20 are outcome specific whereas factors 21–30 are shared across outcomes): (a) antisocial behaviour; (b) violent crimes; (c) hospital admissions; (d) sexual crimes

Fig. 4 summarizes the posterior distribution of $\sum_{i=1}^{n_{1}+n_{2}}\left|\gamma_{ikj}\right|$, i.e. the *L*_1_-norm of the *j*th column of the loadings matrix, where *γ*_*ikj*_ = *λ*_*ik*,*j*−*k*1_ for *j* > *k*_1_. We see that the norm quickly decreases with *j* for both outcome-specific and shared factors, demonstrating the shrinkage effects of the MGPS prior. Inference on the number of non-negligible factors can be done as described in Section 3.4. If we use m = 0.1, then the median posterior number of non-negligible shared factors is 2 (95% credible interval [2,4]) and the median posterior number of factors specific to outcomes 1–4 is 6 (95% credible interval [4,7]), 4 (95% credible interval [3,5]), 14 (95% credible interval [10,19]) and 11 (95% credible interval [9,14]) respectively.

**Fig. 5:**
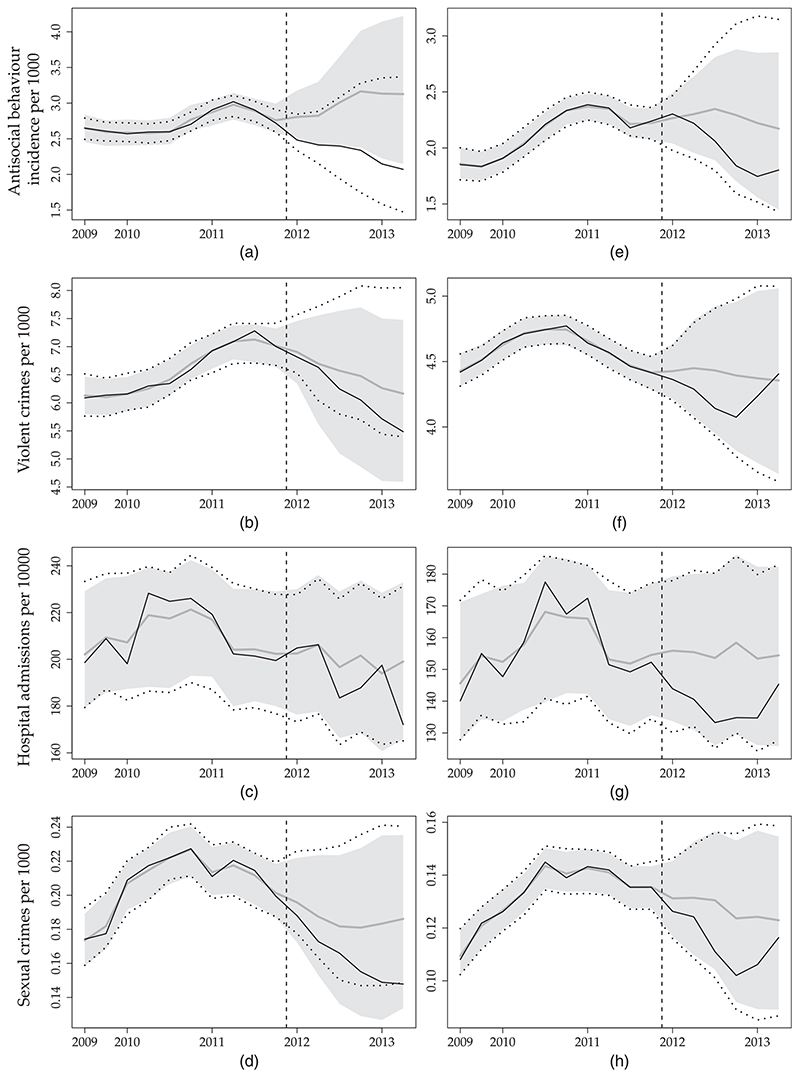
Results of the real data analysis for (a)–(d) Derby and (e)–(h) Enfield 
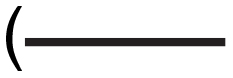
, observed data; 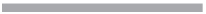, posterior mean of $y_{itk}^{\left(0\right)}$ obtained by fitting MVFA+AR; □, 95% credible intervals of $y_{itk}^{\left(0\right)}$ obtained from the same model;, 95% credible intervals for $y_{itk}^{\left(0\right)}$ obtained by analysing each outcome in turn with the FA model): (a), (e) antisocial behaviour; (b), (f) violent crimes; (c), (g) hospital admissions; (d), (h) sexual crimes

**Fig. 6:**
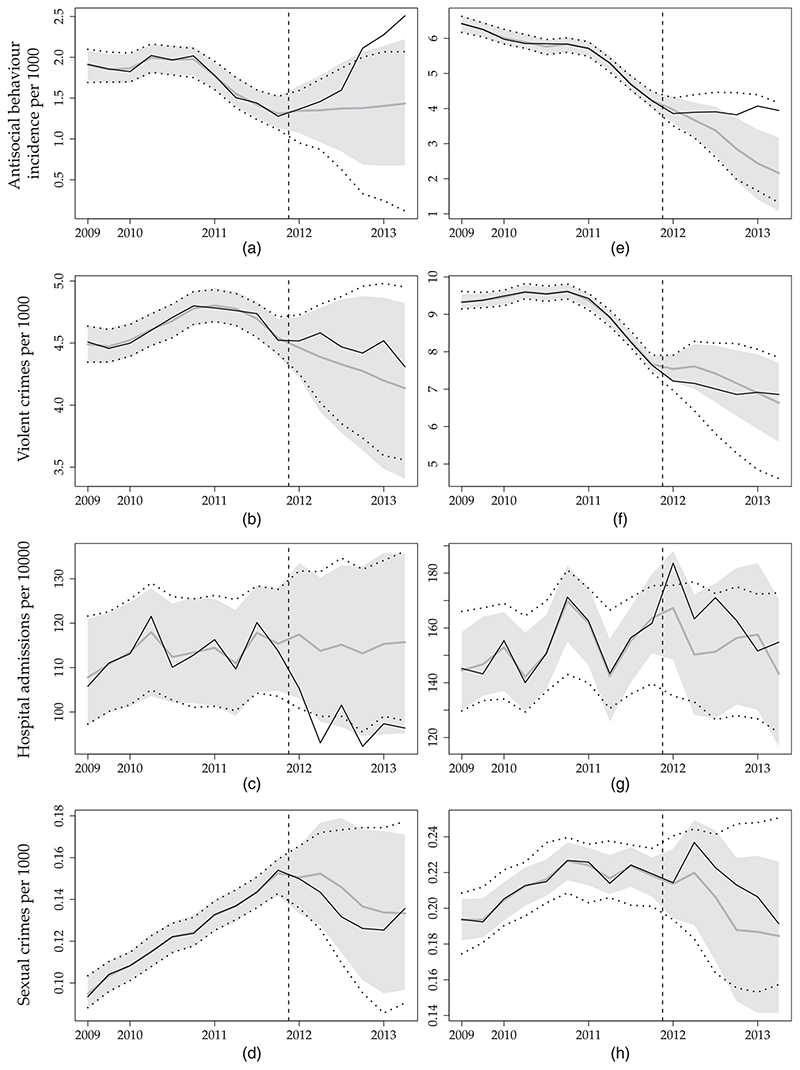
Results of the real data analysis for (a)–(d) Kingston and (e)–(h) Southwark 
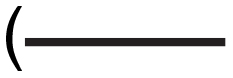
, observed data; 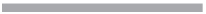, posterior mean of $y_{itk}^{\left(0\right)}$ obtained by fitting MVFA+AR; □, 95% credible intervals of $y_{itk}^{\left(0\right)}$ obtained from the same model;, 95% credible intervals for $y_{itk}^{\left(0\right)}$ obtained by analysing each outcome in turn with the FA model): (a), (e) antisocial behaviour; (b), (f) violent crimes; (c), (g) hospital admissions; (d), (h) sexual crimes

There is not enough evidence in the data to support a significant intervention effect in each unit individually. This is evident in Figs 5 and 6 which show estimated counterfactuals along with their 95% credible intervals for Derby and Enfield, and Kingston and Southwark respectively. We see that, for all treated units and outcomes, the estimated counterfactuals do not appear to be systematically higher than observed values. Further, the 95% credible intervals of the counterfactuals contain the observed values *y_itk_* for most of the combinations of *i* (*i* > 72), *t* (*t*>10) and *k*. This suggests that the data are compatible with what would have been observed if intervention had not taken place.

The point estimates of *ϑ*_*tk*_, the average (over units) intervention effect, for all post-intervention time points 11–16 and outcomes, along with their 95% credible intervals, are shown in Table 2.

We see that the credible intervals for antisocial behaviour, violent crimes and sexual crimes are nearly symmetrical about zero. Therefore we conclude that there is no evidence for an effect of the intervention on these outcomes. For hospital admissions, the point estimates are all negative (a negative value means that admissions would be higher with no intervention). One of the advantages of the Bayesian approach is that it enables us to estimate many interesting causal quantities directly from the posterior distribution of the counterfactuals. Here we focus on the probability that *ϑ*_*tk*_ >0, which for hospital admissions and time points 11–16 is 0.41, 0.18, 0.16, 0.04, 0.10 and 0.07 respectively. Some of these values are low, suggesting that the intervention succeeded in reducing the rate of hospital admissions. However, most of them are higher than 5% and therefore the evidence is inconclusive

## 6 Discussion

In this work, we have introduced the model MVFA+AR for evaluating the effect of a dichotomous intervention from time series observational data. Our model extends in two ways the FA model that is frequently used for causal inference in this setting. First, it models multiple correlated outcomes jointly. Second, it accounts for auto-correlation within each of the outcomes. Both of these extensions enable more efficient estimation of the effect of an intervention on all, or any one, of the multiple outcomes. An important facet of the model proposed is that it provides posterior credible intervals for the causal effects of interest that account for the uncertainty about the number of factors.

The ability to make inference is inherent in the Bayesian framework and therefore in our method. This gives it an advantage over many existing approaches for causal inference using time series observational data, including frequentist approaches based on the FA model (Gobillon and Magnac, 2016; Chan and Kwok, 2016; Xu, 2017; Li, 2018) and synthetic control-type approaches (Abadie *et al*., 2010; Hsiao *et al*., 2012; Doudchenko and Imbens, 2016; Ben-Michael *et al*., 2018). The reason is that, to allow for inference, these methods require assumptions that might be unlikely to hold in some applications and therefore may yield confidence intervals that do not reflect the true uncertainty in the estimates of the causal effect. For example, the parametric bootstrap approach of Xu (2017) relies on the assumption that the error terms in the FA model are homoscedastic at each time point. For the approaches of Abadie *et al*. (2010) and Hsiao *et al*. (2012), inference is typically done with a ‘placebo test’: a procedure that is akin to a permutation test. However, the validity of this test is debatable unless we are willing to assume that the unit that received the intervention was chosen at random (Ben-Michael *et al*., 2018). These assumptions are not essential for our method, suggesting that it can be a useful alternative in applications where they are unlikely to hold.

Our simulation studies indicate that the estimates of intervention effects obtained from MVFA+AR are more precise than those obtained from the standard FA model. This can lead to considerable gains in power for detecting an intervention effect. Further, we found that MVFA+AR has better type I error rate control compared with the standard FA model. Both these gains occur when either the total number of preintervention time points or the total number of control units is relatively small. This is so in many practical problems.

We applied our methodology to estimate the effect of CIPs on alcohol-related harms. We found evidence for the existence of common factors driving the outcomes that we considered. We identified no major effect of CIPs on the rate of antisocial behaviour incidents, violent crimes or sexual crimes. The analysis provides some evidence that the intervention has led to a decrease in the rate of alcohol-related hospital admissions. However, the effect is not significant, i.e. the 95% credible intervals contain zero.

There are limitations to the method proposed. Our model allows for loadings that are shared across all outcomes. However, with K>2 outcomes there is the possibility that there are loadings which are shared between only *k* of them, where 2 ≤ *k* ≤ *K* − 1. There may be a benefit in extending the model to allow for loadings that are common only to a subset of the outcomes. Another extension that may be useful would replace the AR(1) structure that we assume with a more general auto-regressive moving average process. However, this may not be feasible, given the length of the time series that is encountered in many practical applications.

Several possible directions for future research exist. The model proposed does not make use of the geographical location of the units. Such information may be of value, since we expect the outcomes of units with spatial proximity to be correlated. It may be worth extending the model to account for this. Lopes *et al*. (2008) achieved this by assuming a spatial model for the loadings. Finally, although our model should perform well when the normality assumption holds approximately, it cannot be used when the data drastically deviate from this assumption, e.g. when the outcomes are dichotomous. Therefore, it is important to develop a model for mixed outcomes. We shall consider such extensions in our future research.

## Supplementary Material

Supplementary material

## Figures and Tables

**Table 1:** Results of the simulation study for the first outcome *k* = 1 and post-intervention time points *t* = *T*_1_ +1 and *t* = *T* †

Model	Results for the following set-ups:								
	I	II	III	IV	V	VI	VII	VIII	IX
	*T*_1_								
	40	40	40	20	20	20	10	10	10
	*n*_1_								
	30	15	5	30	15	5	30	15	5
*Results for* k=*1 and* t=T_1_ +*1*									
Bias									
MVFA+AR	0.017	0.030	0.119	0.034	0.056	0.155	0.072	0.103	0.195
MVFA	0.035	0.075	0.327	0.055	0.105	0.332	0.100	0.158	0.357
FA+AR	0.026	0.043	0.126	0.065	0.090	0.166	0.126	0.146	0.209
FA	0.044	0.087	0.324	0.082	0.131	0.330	0.144	0.186	0.351
*Standard error*									
MVFA+AR	0.314	0.354	0.480	0.347	0.388	0.508	0.396	0.440	0.539
MVFA	0.322	0.383	0.684	0.355	0.418	0.669	0.406	0.470	0.666
FA+AR	0.330	0.371	0.492	0.387	0.424	0.526	0.460	0.494	0.569
FA	0.335	0.398	0.691	0.392	0.449	0.677	0.466	0.517	0.680
*Mean credible interval width*									
MVFA+AR	1.245	1.394	1.904	1.370	1.548	2.055	1.628	1.833	2.286
MVFA	1.262	1.471	2.429	1.386	1.618	2.450	1.653	1.912	2.614
FA+AR	1.309	1.465	1.934	1.539	1.707	2.104	1.902	2.045	2.357
FA	1.317	1.517	2.425	1.532	1.736	2.462	1.898	2.076	2.634
*False positive rate*									
MVFA+AR	0.050	0.047	0.052	0.053	0.051	0.051	0.048	0.046	0.050
MVFA	0.054	0.055	0.085	0.059	0.058	0.088	0.051	0.056	0.074
FA+AR	0.048	0.049	0.052	0.051	0.049	0.057	0.049	0.050	0.057
FA	0.052	0.059	0.090	0.058	0.064	0.089	0.054	0.062	0.081
*Results for* k =*1 and t =T Bias*									
MVFA+AR	0.014	0.032	0.208	0.043	0.074	0.263	0.113	0.170	0.333
MVFA	0.035	0.084	0.370	0.069	0.133	0.409	0.152	0.241	0.481
FA+AR	0.033	0.057	0.217	0.100	0.140	0.282	0.225	0.259	0.357
FA	0.054	0.107	0.372	0.121	0.186	0.420	0.246	0.308	0.490
*Standard error*									
MVFA+AR	0.326	0.375	0.676	0.374	0.437	0.737	0.484	0.569	0.800
MVFA	0.331	0.398	0.780	0.383	0.467	0.825	0.496	0.606	0.877
FA+AR	0.355	0.414	0.703	0.465	0.538	0.784	0.640	0.703	0.861
FA	0.358	0.433	0.800	0.469	0.554	0.858	0.642	0.717	0.913
*Mean credible interval width*									
MVFA+AR	1.284	1.485	2.483	1.485	1.746	2.660	1.913	2.216	2.931
MVFA	1.283	1.498	2.456	1.459	1.714	2.533	1.874	2.163	2.789
FA+AR	1.392	1.607	2.518	1.812	2.052	2.722	2.454	2.626	3.023
FA	1.373	1.574	2.462	1.728	1.920	2.570	2.341	2.471	2.863
*False positive rate*									
MVFA+AR	0.050	0.050	0.066	0.049	0.049	0.078	0.052	0.058	0.084
MVFA	0.055	0.062	0.125	0.060	0.076	0.147	0.067	0.086	0.141
FA+AR	0.052	0.054	0.076	0.053	0.063	0.089	0.065	0.072	0.099
FA	0.057	0.074	0.133	0.070	0.091	0.159	0.082	0.102	0.155

† The table presents the bias of the point estimates of ϑ_*t*__1_, the standard error of the point estimates, the mean width of the 95% credible intervals and the false positive rate. All results are based on 10000 simulated data sets from MVFA+AR.

**Table 2:** Estimated average (over units) treatment effect for each outcome and post-intervention time point (with 95% posterior credible intervals in parentheses) and average (over units) observed values for each outcome and post-intervention time point

t	Results for antisocial behaviour	Results for violent crimes	Results for hospital admissions	Results for sexual crimes
*Estimates of* ϑ_*tk*_				
11	–0.09 [–0.26, 0.07]	–0.1 [–0.29, 0.09]	–1.2 [–13.1, 9.8]	–0.003 [–0.013, 0.007]
12	–0.04 [–0.27, 0.19]	–0.12 [–0.44, 0.21]	–5.6 [–18.6, 6.7]	–0.003 [–0.018, 0.011]
13	–0.04 [–0.36, 0.28]	–0.22 [–0.63, 0.23]	–6.9 [–20.9, 7.2]	–0.008 [–0.026, 0.011]
14	0.11 [–0.32, 0.52]	–0.22 [–0.7, 0.27]	–12.9 [–27.9, 1.2]	–0.008 [–0.028,0.012]
15	0.26 [–0.19, 0.73]	–0.09 [–0.6, 0.45]	–9.8 [–25.1, 5]	–0.01 [–0.031,0.012]
16	0.36 [–0.12, 0.86]	–0.06 [–0.58, 0.49]	–11.2 [–26.6, 3.2]	–0.009 [–0.029, 0.013]
*Mean observed values*				
11	2.50	5.73	159.4	0.170
12	2.50	5.66	150.8	0.169
13	2.49	5.47	147.3	0.158
14	2.53	5.35	144.4	0.149
15	2.56	5.35	145.3	0.147
16	2.58	5.26	142.2	0.148
