# Supplementary material for: A Bayesian multivariate factor analysis model for evaluating an intervention by using observational time series data on multiple outcomes
Source: J R Stat Soc Ser A Stat Soc. Author manuscript; Available in PMC 2021 Dec 22. (PMC7612111; doi:10.1111/rssa.12569)
Supplement: Supplementary material [file EMS140492-supplement-Supplementary_material.pdf]

Web-based supplementary materials for "**A Bayesian multivariate factor analysis model for evaluating an intervention using observational time-series data on multiple outcomes**" by Samartsidis, Seaman, Montagna, Charlett, Hickman and De Angelis

A Sampling algorithm details

In this section we present the details of the MCMC algorithm that we use in order to obtain samples for the posterior distribution of the MVFA model of Equations (7), (8), (10) and (11). The parameters in the proposed Gibbs algorithm are drawn in blocks, either from their full conditional distributions or using Metropolis-Hastings steps. A sketch of the sampler can be found in Algorithm 1. The details for all steps involved are given in the following Subsections A.1-A.6. Throughout the section we let $p = p_1 + p_2$ be the total number of factors per outcome.

Input **:** Total number of MCMC iterations L

for $\ell = 1$ *to* $\ell = L$ **do**

- Update $\boldsymbol{\beta}_k$ for all k (Gibbs)
- Update $\boldsymbol{f}_k = \left(\boldsymbol{s}_{1k}^\top, \boldsymbol{f}_{1k}^\top, \ldots, \boldsymbol{s}_{Tk}^\top, \boldsymbol{f}_{Tk}^\top\right)^\top$ for all k (Gibbs)
- Update ρ_{jk} for all j and k (Metropolis-Hastings)
- Update $\tilde{\boldsymbol{\lambda}}_i = \left(\boldsymbol{\lambda}_i^\top, \boldsymbol{\gamma}_{i1}^\top, \ldots, \boldsymbol{\gamma}_{iK}^\top\right)^\top$ for all i (Gibbs)
- Update ϕ_{ikj} for all i, k and j (Gibbs)
- Update δ_{kj} for all k and j (Gibbs)
- Update ψ_{ik}^2 for all i and k (Gibbs)
- Save the current state of all model parameters to $\boldsymbol{\theta}^{(\ell)}$

end

Output: $\boldsymbol{\theta}^{(1)}, \ldots, \boldsymbol{\theta}^{(L)}$

Algorithm 1: Hybrid Gibbs sampler for the multivariate factor analysis model with an AR(1) prior on the factors and a multiplicative Gamma process shrinkage prior on the loadings.

A.1 Update of regression coefficients

Let P be the total number of covariates. For each outcome k and time t, let $\boldsymbol{X}_{tk}$ denote the $n \times P$ matrix with rows $\boldsymbol{x}_{it}$. We have that:

$$\begin{aligned}
\pi\left(\boldsymbol{\beta}_k \mid \text{rest}\right) &\propto \mathrm{N}\left(\boldsymbol{\beta}_k; \boldsymbol{0}, 10^3 \boldsymbol{I}\right) \prod_{t=1}^{T} \mathrm{N}\left(\boldsymbol{y}_{tk}; \boldsymbol{\Gamma}_k \boldsymbol{s}_{tk} + \boldsymbol{\Lambda} \boldsymbol{f}_{tk} + \boldsymbol{X}_{tk}\boldsymbol{\beta}_k, \boldsymbol{\Psi}_k\right) \\
&\propto \exp\left(\sum_{t=1}^{T}\left[\tilde{\boldsymbol{y}}_{tk}^\top \boldsymbol{\Psi}_k^{-1} \boldsymbol{X}_{tk}\right]\boldsymbol{\beta}_k - \frac{1}{2}\boldsymbol{\beta}_k^\top \sum_{t=1}^{T}\left[\boldsymbol{X}_{tk}^\top \boldsymbol{\Psi}_k^{-1} \boldsymbol{X}_{tk} + 10^{-3}\boldsymbol{I}\right]\boldsymbol{\beta}_k\right),
\end{aligned}$$

where $\tilde{\boldsymbol{y}}_{tk} = \boldsymbol{y}_{tk} - \boldsymbol{\Gamma}_k \boldsymbol{s}_{tk} - \boldsymbol{\Lambda} \boldsymbol{f}_{tk}$. Hence, we draw $\boldsymbol{\beta}_k$ from a multivariate normal distribution with variance-covariance $\boldsymbol{J}_k = \left(\sum_{t=1}^{T}\left[\boldsymbol{X}_{tk}^\top \boldsymbol{\Psi}_k^{-1} \boldsymbol{X}_{tk} + 10^{-3}\boldsymbol{I}\right]\right)^{-1}$ and mean $\boldsymbol{h}_k = \boldsymbol{J}_k \sum_{t=1}^{T}\left[\boldsymbol{X}_{tk}^\top \boldsymbol{\Psi}_k^{-1} \tilde{\boldsymbol{y}}_{tk}\right]$. For the remainder of this section, we will omit the covariates in order to ease the notation. However, the updates that we describe can be used for the MVFA model with covariates. In particular, they can be used as shown by replacing y_{itk} with $y_{itk} - \boldsymbol{x}_{it}^\top \boldsymbol{\beta}_k$.

A.2 Update of factors

For each outcome k, we define $\boldsymbol{F}_k$ as the $T \times (p_1 + p_2)$ matrix with elements

$$(\boldsymbol{F}_k)_{tj} = \begin{cases} s_{tjk} & j \leq p_1 \\ f_{t,j-p_1,k} & j > p_1 \end{cases}. \tag{1}$$

Let $\boldsymbol{f}^r_{tk}$ be the t-th row of $\boldsymbol{F}_k$ $(t = 1, \dots, T)$ and $\boldsymbol{f}^c_{jk}$ be the j-th column of $\boldsymbol{F}_k$ $(j = 1, \dots, p)$. For each j, we have assumed that $\boldsymbol{f}^c_{jk}$ arises from an AR(1) process with persistent parameter ρ_{jk}. Therefore, for each j we *a priori* have that $\left[\boldsymbol{f}^c_{jk} \mid \rho_{jk}\right] \sim \mathrm{N}_T\left(\boldsymbol{0}, \boldsymbol{Q}^{-1}_{jk}\right)$ where $\boldsymbol{0}$ is a T-vector of zeroes and

$$\boldsymbol{Q}_{jk} = \begin{bmatrix} 1 & -\rho_{jk} & 0 & \cdots & 0 \\ -\rho_{jk} & (1+\rho^2_{jk}) & -\rho_{jk} & & \vdots \\ 0 & -\rho_{jk} & \ddots & \ddots & 0 \\ \vdots & \ddots & \ddots & (1+\rho^2_{jk}) & -\rho_{jk} \\ 0 & \cdots & 0 & -\rho_{jk} & 1 \end{bmatrix}, \tag{2}$$

see e.g. Kastner and Frühwirth-Schnatter (2014). Let $\boldsymbol{f}_k = \left(\boldsymbol{f}^{r\top}_{1k}, \dots, \boldsymbol{f}^{r\top}_{Tk}\right)^\top$. Equation (2) implies that $[\boldsymbol{f}_k \mid \boldsymbol{\rho}_k] \sim \mathrm{N}\left(\boldsymbol{0}, \boldsymbol{Q}^{-1}_k\right)$; here, $\boldsymbol{\rho}_k = (\rho_{1k}, \dots, \rho_{pk}, \dots, \rho_{1k}, \dots, \rho_{pk})$ and $\boldsymbol{Q}_k$ is a block-tridiagonal $pT \times pT$ matrix of the form

$$\boldsymbol{Q}_k = \begin{bmatrix} \boldsymbol{Q}_{k1} & \boldsymbol{Q}_{k2} & \boldsymbol{0} & \cdots & \boldsymbol{0} \\ \boldsymbol{Q}_{k2} & \boldsymbol{Q}_{k3} & \boldsymbol{Q}_{k2} & & \vdots \\ \boldsymbol{0} & \boldsymbol{Q}_{k2} & \ddots & \ddots & \\ \vdots & \ddots & \ddots & \boldsymbol{Q}_{k3} & \boldsymbol{Q}_{k2} \\ \boldsymbol{0} & \cdots & \boldsymbol{0} & \boldsymbol{Q}_{k2} & \boldsymbol{Q}_{k1} \end{bmatrix}, \tag{3}$$

and the $p \times p$ blocks are $\boldsymbol{Q}_{k1} = \mathrm{diag}\,\{1, \dots, 1\}$, $\boldsymbol{Q}_{k2} = \mathrm{diag}\,\{-\rho_{1k}, \dots, -\rho_{pk}\}$ and $\boldsymbol{Q}_{k3} = \mathrm{diag}\left\{(1+\rho^2_1), \dots, (1+\rho^2_{pk})\right\}$. Let $\tilde{\boldsymbol{\Lambda}}_k = [\boldsymbol{\Gamma}_k, \boldsymbol{\Lambda}]$. The full conditional of $\boldsymbol{f}_k$ is

$$\begin{aligned}
\pi(\boldsymbol{f}_k \mid \text{rest}) &\propto \mathrm{N}\left(\boldsymbol{f}_k; \boldsymbol{0}, \boldsymbol{Q}^{-1}_k\right) \prod_{t=1}^{T} \mathrm{N}\left(\boldsymbol{y}_{tk}; \tilde{\boldsymbol{\Lambda}}_k \boldsymbol{f}^r_{tk}, \boldsymbol{\Psi}_k\right) \\
&\propto \mathrm{N}\left(\boldsymbol{f}_k; \boldsymbol{0}, \boldsymbol{Q}^{-1}_k\right) \prod_{t=1}^{T} \exp\left(-\frac{1}{2}(\boldsymbol{y}_{tk} - \tilde{\boldsymbol{\Lambda}}_k \boldsymbol{f}^r_{tk})^\top \boldsymbol{\Psi}^{-1}_k (\boldsymbol{y}_{tk} - \tilde{\boldsymbol{\Lambda}}_k \boldsymbol{f}^r_{tk})\right) \\
&\propto \exp\left(-\frac{1}{2}\boldsymbol{f}^\top_k \boldsymbol{Q}_k \boldsymbol{f}_k\right) \exp\left(\sum_{k=1}^{p}\left[\boldsymbol{y}^\top_{tk} \boldsymbol{\Psi}^{-1}_k \tilde{\boldsymbol{\Lambda}}_k \boldsymbol{f}^r_{tk} - \frac{1}{2}\boldsymbol{f}^{r\top}_{tk} \tilde{\boldsymbol{\Lambda}}^\top_k \boldsymbol{\Psi}^{-1}_k \tilde{\boldsymbol{\Lambda}}_k \boldsymbol{f}^r_{tk}\right]\right) \\
&= \exp\left(\boldsymbol{b}^{*\top}_k \boldsymbol{f}_k - \frac{1}{2}\boldsymbol{f}^\top_k \boldsymbol{Q}^*_k \boldsymbol{f}_k\right),
\end{aligned} \tag{4}$$

where $\boldsymbol{b}^*_k = \left(\left(\tilde{\boldsymbol{\Lambda}}^\top_k \boldsymbol{\Psi}^{-1}_k \boldsymbol{y}_{1k}\right)^\top, \dots, \left(\tilde{\boldsymbol{\Lambda}}^\top_k \boldsymbol{\Psi}^{-1}_k \boldsymbol{y}_{Tk}\right)^\top\right)^\top$ and $\boldsymbol{Q}^*_k = \boldsymbol{Q}_k + \mathrm{diag}\left\{\tilde{\boldsymbol{\Lambda}}^\top_k \boldsymbol{\Psi}^{-1}_k \tilde{\boldsymbol{\Lambda}}_k, \dots, \tilde{\boldsymbol{\Lambda}}^\top_k \boldsymbol{\Psi}^{-1}_k \tilde{\boldsymbol{\Lambda}}_k\right\}$. Sampling $\boldsymbol{f}_k$ from its normal full conditional (4) can be done using Algorithm 2 proposed by Rue (2001). Despite the dimensionality of $\boldsymbol{f}_k$, the algorithm can be implemented with great computational efficiency due the block-triagonal structure of $\boldsymbol{Q}^*_k$ which allows for fast calculation of the Cholesky decomposition.

A.3 Update of persistent parameters

Let f_{tjk} be the j-th factor at time t and outcome k, where $j = 1, \dots, p = p_1 + p_2$ and $f_{tjk} = s_{tjk}$ for $j > p_1$. The initial values f_{0jk} can be drawn from their full conditional $\mathrm{N}\left(\rho_{jk} f_{1jk}, 1\right)$ distribution

Input : $\boldsymbol{b}$ and $\boldsymbol{Q}$

Find $\boldsymbol{L}$ such that $\boldsymbol{Q} = \boldsymbol{L}\boldsymbol{L}^\top$;

Find $\boldsymbol{v}$ that solves $\boldsymbol{L}\boldsymbol{v} = \boldsymbol{b}$;

Find $\boldsymbol{\mu}$ that solves $\boldsymbol{L}^\top \boldsymbol{\mu} = \boldsymbol{v}$;

Draw $\boldsymbol{z} \sim \mathrm{N}_d(\boldsymbol{0}, \boldsymbol{I})$;

Find $\boldsymbol{w}$ that solves $\boldsymbol{L}^\top \boldsymbol{w} = \boldsymbol{z}$;

Set $\boldsymbol{x} = \boldsymbol{\mu} + \boldsymbol{w}$;

Output: $\boldsymbol{x} \sim \pi(\boldsymbol{x})$

Algorithm 2: Sampling from a normal distribution of the form $\pi(\boldsymbol{x}) \propto \exp\left(\boldsymbol{b}^\top \boldsymbol{x} - \frac{1}{2}\boldsymbol{x}^\top \boldsymbol{Q}\boldsymbol{x}\right)$, where $\boldsymbol{x}$ is of dimension d. Algorithm proposed by Rue (2001).

(Kastner and Frühwirth-Schnatter, 2014). As noted by Kastner and Frühwirth-Schnatter (2014), the full conditional of the persistent parameter ρ_{jk} is

$$\begin{aligned}
\pi(\rho_{jk} \mid \text{rest}) &\propto \pi(\rho_{jk})\pi(f_{0jk} \mid \rho_{jk}) \prod_{t=1}^{T} \mathrm{N}\left(f_{tjk}; \rho_{jk} f_{t-1,jk}, 1\right) \\
&\propto \mathbb{I}\left(\rho_{jk} \in (-1,1)\right) \pi(f_{0jk} \mid \rho_{jk}) \prod_{t=1}^{T} \exp\left(-\frac{1}{2}(f_{tjk} - \rho_{jk} f_{t-1,j,k})^2\right) \\
&\propto \mathbb{I}\left(\rho_{jk} \in (-1,1)\right) \pi(f_{0jk} \mid \rho_{jk}) \mathrm{N}\left(\rho_{jk}; \frac{\sum_{t=1}^{T} f_{t-1,jk} f_{tjk}}{\sum_{t=0}^{T-1} f_{tjk}^2}, \frac{1}{\sum_{t=0}^{T-1} f_{tjk}^2}\right),
\end{aligned}$$

where $[f_{0jk} \mid \rho_{jk}] \sim \mathrm{N}\left(0, \frac{1}{1-\rho_{jk}^2}\right)$. Therefore we draw a new value $\rho_{jk}^* \sim \mathrm{N}\left(\rho_{jk}^*; \frac{\sum_{t=1}^{T} f_{t-1,jk} f_{tjk}}{\sum_{t=0}^{T-1} f_{tjk}^2}, \frac{1}{\sum_{t=0}^{T-1} f_{tjk}^2}\right)$ and accept this value with probability $\min\left\{1, \mathbb{I}\left(\rho_{jk}^* \in (-1,1)\right) \frac{\pi(f_{0jk} \mid \rho_{jk}^*)}{\pi(f_{0jk} \mid \rho_{jk})}\right\}$.

A.4 Update of loadings

For each unit i we define $\tilde{\boldsymbol{\lambda}}_{ik} = \left(\boldsymbol{\gamma}_{ik}^\top, \boldsymbol{\lambda}_i^\top\right)^\top$, where $\boldsymbol{\gamma}_{ik}$ and $\boldsymbol{\lambda}_i$ are the i-th rows of $\boldsymbol{\Gamma}_k$ and $\boldsymbol{\Lambda}$, respectively. Further, let $\boldsymbol{\Phi}_{ik} = \operatorname{diag}\left\{\frac{1}{\phi_{i1k}^0 \tau_{1k}^0}, \ldots, \frac{1}{\phi_{ip_1k}^0 \tau_{p_1k}^0}, \ldots, \frac{1}{K\phi_{i1k}^1 \tau_{1k}^1}, \ldots, \frac{1}{K\phi_{ip_2k}^1 \tau_{p_2k}^1}\right\}$, where we have used superscripts 0 and 1 to denote the MGPS shrinkage parameters of the outcome-specific and shared loadings, respectively. Note that in $\boldsymbol{\Phi}_{ik}$ we have divided the MGPS parameters of the shared loadings by K because their prior contributes to all K outcomes. For each outcome k, we have that

$$\begin{aligned}
\pi(\tilde{\boldsymbol{\lambda}}_{ik} \mid \text{rest}) &\propto \mathrm{N}\left(\tilde{\boldsymbol{\lambda}}_{ik}; \boldsymbol{0}_p, \boldsymbol{\Phi}_{ik}\right) \mathrm{N}\left(\boldsymbol{y}_{ik}; \boldsymbol{F}_k \tilde{\boldsymbol{\lambda}}_{ik}, \psi_{ik}^2 \boldsymbol{I}\right) \\
&\propto \exp\left(\boldsymbol{y}_{ik}^\top \boldsymbol{\Sigma}_{ik}^{-1} \boldsymbol{F}_k \tilde{\boldsymbol{\lambda}}_{ik} - \frac{1}{2}\tilde{\boldsymbol{\lambda}}_{ik}^\top \left(\boldsymbol{F}_k^\top \boldsymbol{\Sigma}_{ik}^{-1} \boldsymbol{F}_k + \boldsymbol{\Phi}_{ik}^{-1}\right) \tilde{\boldsymbol{\lambda}}_{ik}\right) \\
&= \exp\left(\boldsymbol{w}_{ik}^\top \tilde{\boldsymbol{\lambda}}_{ik} - \frac{1}{2}\tilde{\boldsymbol{\lambda}}_{ik}^\top \boldsymbol{G}_{ik} \tilde{\boldsymbol{\lambda}}_{ik}\right), \qquad (5)
\end{aligned}$$

where $\boldsymbol{F}_k$ was defined is Section A.2 and $\boldsymbol{\Sigma}_{ik} = \psi_{ik}^2 \boldsymbol{I}$. We first write $\boldsymbol{w}_{ik} = \left(\boldsymbol{w}_{ik}^{1\,\top}, \boldsymbol{w}_{ik}^{2\,\top}\right)^\top$ and $\boldsymbol{G}_{ik} = \begin{bmatrix} \boldsymbol{G}_{ik}^1 & \boldsymbol{G}_{ik}^2 \\ \boldsymbol{G}_{ik}^{2\,\top} & \boldsymbol{G}_{ik}^3 \end{bmatrix}$, where $\boldsymbol{w}_{ik}^1$ is a p_1-vector, $\boldsymbol{w}_{ik}^1$ is a p_2-vector, $\boldsymbol{G}_{ik}^1$ is a $p_1 \times p_1$ matrix, $\boldsymbol{G}_{ik}^2$ is a $p_1 \times p_2$ matrix and $\boldsymbol{G}_{ik}^3$ is a $p_2 \times p_2$ matrix. Let $\tilde{\boldsymbol{\lambda}}_i = (\boldsymbol{\gamma}_{i1}, \ldots, \boldsymbol{\gamma}_{iK}, \boldsymbol{\lambda}_i)$. Equation 5 implies that

$$\pi(\tilde{\boldsymbol{\lambda}}_i \mid \text{rest}) \propto \exp\left(\boldsymbol{w}_i^\top \tilde{\boldsymbol{\lambda}}_i - \frac{1}{2}\tilde{\boldsymbol{\lambda}}_i^\top \boldsymbol{G}_i \tilde{\boldsymbol{\lambda}}_i\right),$$

that is, $\tilde{\boldsymbol{\lambda}}_i$ *a posteriori* has a normal distribution with variance-covariance matrix $\boldsymbol{G}_i^{-1}$ and mean $\boldsymbol{G}_i^{-1}\boldsymbol{w}_i$, where $\boldsymbol{w}_i = \left(\boldsymbol{w}_{i1}^{1\top}, \ldots, \boldsymbol{w}_{iK}^{1\top}, \sum_{k=1}^{K} \boldsymbol{w}_{ik}^{2\top}\right)$ and

$$\boldsymbol{G}_i = \begin{bmatrix} \boldsymbol{G}_{i1}^1 & \boldsymbol{0} & \ldots & \ldots & \boldsymbol{G}_{i1}^2 \\ \boldsymbol{0} & \boldsymbol{G}_{i2}^1 & \boldsymbol{0} & \ldots & \boldsymbol{G}_{i2}^2 \\ \vdots & \boldsymbol{0} & \ddots & & \vdots \\ \vdots & \vdots & & \ddots & \boldsymbol{G}_{iK}^2 \\ \boldsymbol{G}_{i1}^{2\top} & \boldsymbol{G}_{i2}^{2\top} & \ldots & \boldsymbol{G}_{iK}^{2\top} & \sum_{k=1}^{K} \boldsymbol{G}_{ik}^3 \end{bmatrix}.$$

We use Algorithm 2 to simulate from $\pi(\tilde{\boldsymbol{\lambda}}_i \mid \text{rest})$ and update the loadings.

A.5 Update of shrinkage parameters

As shown by Bhattacharya and Dunson (2011), the full conditional distributions of the MGPS prior parameters are available in closed form. Here, we list them for outcome-specific loadings. In particular, for each unit i, factor $j \leq p_1$ and outcome k, we have that

$$[\phi_{ijk} \mid \text{rest}] \sim \text{Gamma}\left(\frac{\nu+1}{2}, \frac{\nu + \tau_{jk}\lambda_{ijk}^2}{2}\right).$$

Moreover, for each outcome k we have that for factor $j = 1$

$$[\delta_{1k} \mid \text{rest}] \sim \text{Gamma}\left(\alpha_1 + \frac{nk_1}{2}, \frac{1}{2}\sum_{\ell=1}^{k_1} \omega_\ell^{(1)} \sum_{i=1}^{n} \phi_{i\ell k}\lambda_{i\ell k}^2\right),$$

and for factors j, $2 \leq j \leq p_1$, that

$$[\delta_{jk} \mid \text{rest}] \sim \text{Gamma}\left(\alpha_2 + \frac{n}{2}(k_1 - j + 1), \frac{1}{2}\sum_{\ell=j}^{k_1} \omega_\ell^{(j)} \sum_{i=1}^{n} \phi_{i\ell k}\lambda_{i\ell k}^2\right),$$

where $\omega_\ell^j = \prod_{t=1, t\neq j}^{\ell} \delta_t$. Updates for shared loadings are analogous and therefore are not shown.

A.6 Update of variances

The inverse Gamma prior is conjugate for the variance terms ψ_{ik}^2. In particular, for each unit i and outcome k, we have that

$$[\psi_{ik}^2 \mid \text{rest}] \sim \text{IG}\left(\frac{T}{2} + \alpha_0, \frac{1}{2}\sum_{t=1}^{T}\left(y_{itk} - \boldsymbol{f}_{tk}^{r\top}\tilde{\boldsymbol{\lambda}}_{ik}\right)^2 + \beta_0\right).$$

B Supplementary material for simulation studies

In this section, we provide supplementary results for the simulation study of Section 3. Table 1 presents bias, standard deviation, mean credible interval width and false positive rate for $(k, t) = (2, T_1 + 1)$ and $(k, t) = (3, T)$. Figure 1 shows power for $(k, t) = (2, T_1 + 1)$. Finally, Figures 1 shows power for $(k, T) = (3, T)$.

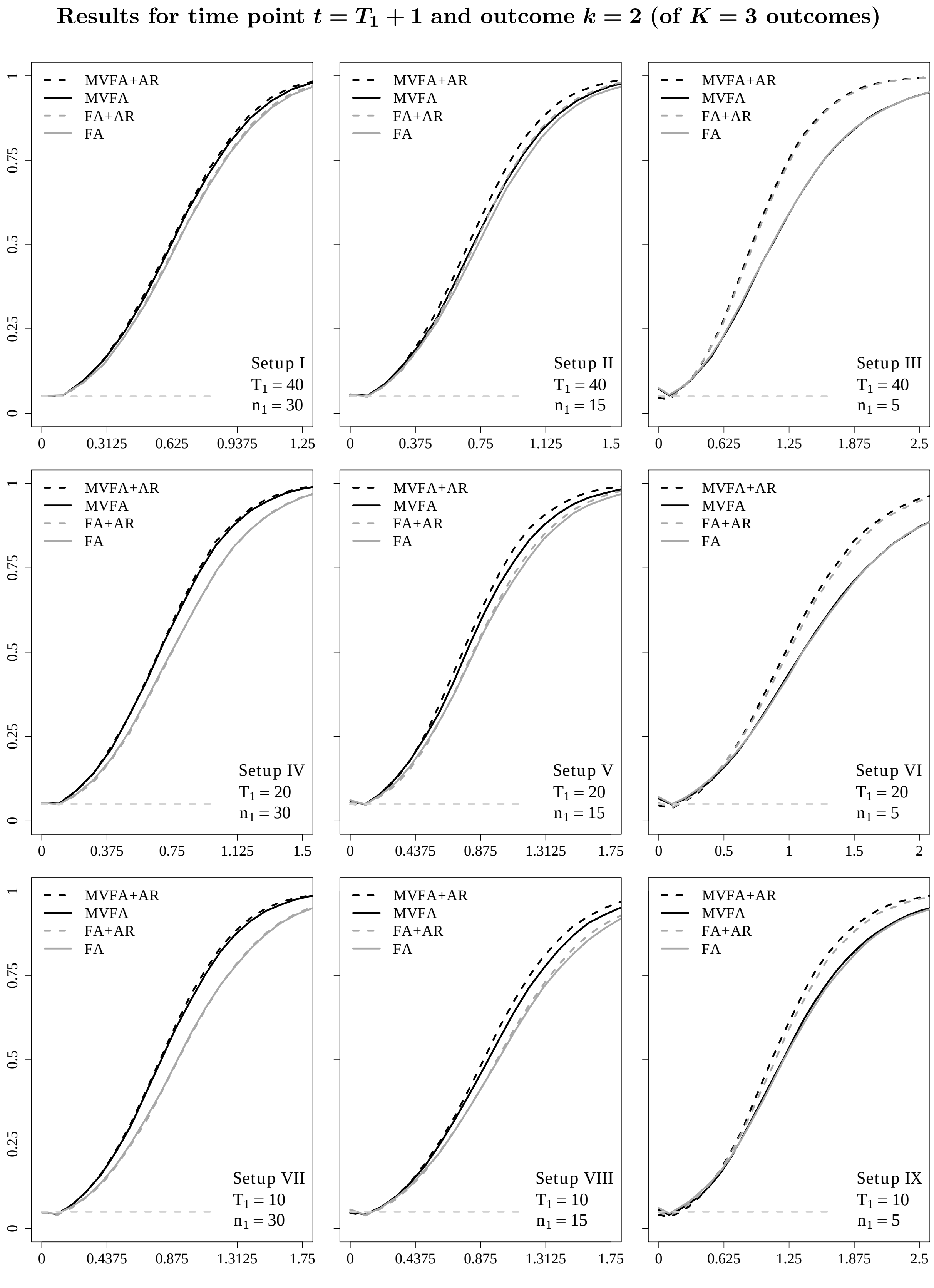

Figure 1: Results of the simulation study for the *second outcome* $k = 2$ and *first post-intervention time point* $t = T_1 + 1$. The figure presents the probability of detecting an intervention effect (y-axis) as a function of $\vartheta_{T_1+1,2}$ (x-axis) in Setups I-IX. All results are based on 10000 datasets simulated from the MVFA+AR model. The horizontal dashed lines indicate 5%, the desired detection rate when the intervention $\vartheta_{T_1+1,2} = 0$.

Results for time point $t = T$ and outcome $k = 3$ (of $K = 3$ outcomes)

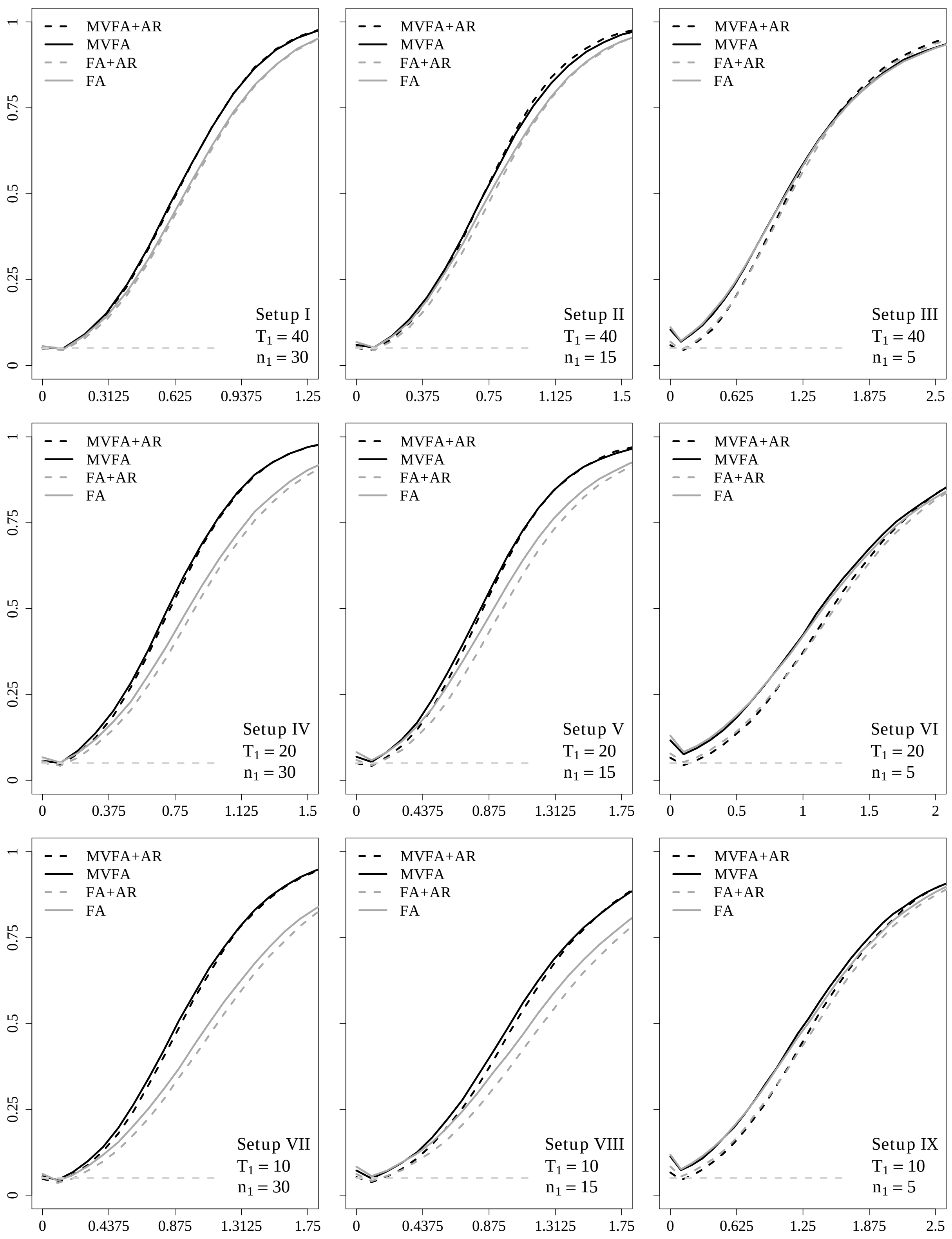

Figure 2: Results of the simulation study for the *third outcome* $k = 3$ and *last post-intervention time point* $t = T$. The figure presents the probability of detecting an intervention effect (y-axis) as a function of $\vartheta_{T,3}$ (x-axis) in Setups I-IX. All results are based on 10000 datasets simulated from the MVFA+AR model. The horizontal dashed lines indicate 5%, the desired detection rate when the intervention $\vartheta_{T,3} = 0$.

References

Bhattacharya, A. and D. B. Dunson (2011). Sparse bayesian infinite factor models. *Biometrika 98*(2), 291–306.

Kastner, G. and S. Frühwirth-Schnatter (2014, August). Ancillarity-sufficiency interweaving strategy (ASIS) for boosting MCMC estimation of stochastic volatility models. *Computational Statistics and Data Analysis 76*(C), 408–423.

Rue, H. (2001). Fast sampling of Gaussian Markov random fields. *Journal of the Royal Statistical Society: Series B (Statistical Methodology) 63*(2), 325–338.

Setup	I	II	III	IV	V	VI	VII	VIII	IX
T_1	40	40	40	20	20	20	10	10	10
n_1	30	15	5	30	15	5	30	15	5

Results for $k = 2$ and $t = T_1 + 1$

**Bias**									
MVFA+AR	0.003	0.004	0.007	0.003	0.003	0.005	0.005	0.004	0.005
MVFA	0.003	0.005	0.006	0.003	0.003	0.003	0.005	0.005	0.008
FA+AR	0.002	0.003	0.007	0.002	0.001	0.004	0.004	0.004	0.006
FA	0.002	0.005	0.007	0.002	0.002	0.004	0.004	0.005	0.005
**Standard error**									
MVFA+AR	0.314	0.351	0.483	0.347	0.389	0.511	0.398	0.445	0.548
MVFA	0.321	0.381	0.685	0.355	0.419	0.674	0.407	0.474	0.669
FA+AR	0.331	0.371	0.495	0.388	0.433	0.531	0.465	0.507	0.575
FA	0.336	0.396	0.688	0.393	0.455	0.681	0.472	0.527	0.681
**Mean credible interval width**									
MVFA+AR	1.237	1.381	1.874	1.362	1.536	2.024	1.620	1.824	2.257
MVFA	1.253	1.454	2.365	1.376	1.603	2.384	1.645	1.899	2.537
FA+AR	1.301	1.450	1.897	1.528	1.692	2.061	1.887	2.026	2.311
FA	1.308	1.503	2.358	1.522	1.718	2.389	1.884	2.054	2.551
**False positive rate**									
MVFA+AR	0.050	0.051	0.046	0.050	0.050	0.046	0.048	0.045	0.040
MVFA	0.051	0.056	0.073	0.053	0.055	0.066	0.048	0.048	0.055
FA+AR	0.051	0.051	0.051	0.052	0.054	0.053	0.047	0.052	0.048
FA	0.051	0.055	0.075	0.054	0.061	0.071	0.050	0.056	0.061

Results for $k = 3$ and $t = T$

**Bias**									
MVFA+AR	-0.000	-0.003	0.001	0.001	0.000	0.004	0.001	0.001	0.005
MVFA	0.000	-0.001	0.004	0.000	-0.001	0.002	0.000	-0.004	0.004
FA+AR	-0.001	-0.004	0.001	-0.001	-0.001	0.004	-0.003	-0.005	0.004
FA	-0.000	-0.003	0.003	-0.001	-0.002	0.003	-0.003	-0.003	0.005
**Standard error**									
MVFA+AR	0.322	0.373	0.668	0.374	0.440	0.729	0.478	0.563	0.795
MVFA	0.328	0.395	0.784	0.382	0.468	0.829	0.490	0.596	0.877
FA+AR	0.351	0.410	0.692	0.468	0.540	0.776	0.628	0.699	0.854
FA	0.355	0.429	0.805	0.470	0.556	0.863	0.631	0.712	0.915
**Mean credible interval width**									
MVFA+AR	1.274	1.465	2.426	1.474	1.723	2.602	1.902	2.194	2.868
MVFA	1.272	1.476	2.393	1.446	1.685	2.459	1.861	2.135	2.709
FA+AR	1.381	1.587	2.454	1.792	2.018	2.647	2.435	2.594	2.939
FA	1.361	1.551	2.393	1.710	1.886	2.484	2.324	2.437	2.770
**False positive rate**									
MVFA+AR	0.049	0.051	0.059	0.051	0.050	0.066	0.048	0.053	0.066
MVFA	0.052	0.060	0.104	0.057	0.069	0.116	0.055	0.072	0.113
FA+AR	0.049	0.055	0.069	0.056	0.059	0.078	0.052	0.062	0.084
FA	0.056	0.068	0.111	0.067	0.082	0.130	0.062	0.083	0.118

Table 1: Results of the simulation study for $(k, t) = (2, T_1+1)$ and $(k, t) = (3, T)$. The table presents the bias of the point estimates of ϑ_{tk}, the standard error of the point estimates, the mean width of the 95% credible intervals and the false positive rate. All results are based on 10000 simulated datasets from the MVFA+AR model.
